# Social Interactions for Sustainable Food Choices: Meeting the Target for Meat Intake in the United Kingdom

**DOI:** 10.1016/j.cdnut.2025.107509

**Published:** 2025-07-30

**Authors:** Angela Fontan, Rosemary Green, Karl H Johansson, Patricia Eustachio Colombo

**Affiliations:** 1 Division of Decision and Control Systems, School of Electrical Engineering and Computer Science, KTH Royal Institute of Technology, Stockholm, Sweden; 2 Centre on Climate Change and Planetary Health, London School of Hygiene and Tropical Medicine, London, United Kingdom; 3 Department of Medicine, Karolinska Institutet, Stockholm, Sweden

**Keywords:** sustainable diets, meat reduction, agent-based modeling, external influence, social networks

## Abstract

**Background:**

Substantial shifts in contemporary diets are needed to halt the growing burden of chronic diseases and the accelerating climate crisis. Achieving this will require strong action and the use of policy instruments that can aid consumers in making the right dietary choices. Real-life evidence on changing dietary behaviors within complex food systems at scale demands resources and research that might not be realistic to acquire or realize.

**Objectives:**

This project applied innovative models to predict the dietary and environmental effects of broad-scale strategies designed to transform dietary behaviors among consumers. Scenarios were numerically simulated using agent-based opinion-dynamics models to evaluate the impact of governmental influence on people’s consumption of meat, vegetables, pulses, and meat alternatives.

**Methods:**

We considered the problem of influencing the opinions of a group of agents (representing the UK population) connected in a social network over a sequence of campaigns to achieve convergence to 2 different targets: achieving the UK Climate Change Committee’s goal of 35% and 50% average reduction in meat consumption by 2030 and 2050, respectively. The opinion dynamics comprised consumers (i.e., agents), with empirically derived baseline food intake patterns (i.e., initial opinions) and sociodemographic attributes. Changes in environmental impacts following changes in consumption were quantified.

**Results:**

In a scenario of high governmental influence, uniform in the population, achievement of the 35% and 50% meat reduction goals corresponded to ∼5.2 and ∼8.1 y, respectively. As meat consumption reduced, intake of vegetables, pulses, and meat alternatives increased by 84.7%, 108.5%, and 30.8%, respectively (−35% scenario). Greenhouse gas emissions, land use, water use, and scarcity-weighted water were reduced by −23.8%, −20.2%, −0.5%, and −14.5%, respectively (−35% scenario).

**Conclusions:**

The findings of this project provide valuable insights for policymakers and governmental authorities to develop effective influence strategies aimed at promoting healthy and sustainable dietary habits.

## Introduction

There is an urgent need to align human diets with goals for environmental sustainability and population health. Imbalanced diets are today’s major contributors to the burden of chronic diseases as well as to greenhouse gas emissions and environmental degradation globally [1,2]. Thus, transitioning to nutritious and sustainable diets [3] will be crucial for meeting the ambitious public health goals and commitments to net-zero emissions in G7 economies such as the European Union and the United Kingdom. In 2019, the EAT–Lancet Commission suggested a global planetary health diet, based on a full scientific review of what constitutes a healthy and nutritious diet from a sustainable food system [1]. It emphasizes a plant-forward diet where meat and dairy constitute important parts of the diet but in significantly smaller proportions than whole grains, fruits, vegetables, nuts, and legumes. Since then, research has been able to demonstrate the health and environmental benefits of reducing the intake of animal products while increasing the consumption of plant-based foods [[4], [5], [6]]. Against this backdrop, countries in the Western world have been setting up food consumption guidelines and dietary targets to promote these shifts [[7], [8], [9]].

Despite these efforts, limited knowledge persists regarding the design of policies to implement the desired changes at the population level and their practical realization. Additionally, there remains uncertainty surrounding how individuals respond to repeated peer or social pressure, as well as external pressures to conform to a desired norm. Reasons for these knowledge gaps arise, for instance, from empirical limitations associated with studying large populations over extended periods. Additionally, at a design level, challenges arise from the inherent difficulties in selecting or formulating a predictive model and adhering to its conventional constraints. Agent-based models (ABMs) are computational or simulation models that describe the behavior of a system composed of heterogeneous agents that influence each other over time, by encoding the behavior of individual agents in communication or interaction protocols [10(p. 22),^11^(p.606)]; see previous studies [10,12] for comprehensive introductions to the topic. ABMs allow researchers to stretch beyond the currently dominating risk factor approach to nutritional epidemiology, as they enable the creation of virtual laboratories in which behaviors of populations can be predicted and investigated using a what if approach [13], for both small-size and large-size communities [14]. In the context of food systems, typical applications of ABMs include investigating food consumption behavior [15], assessing the impact of policies [16], and identifying optimal interventions aimed at, for instance, promoting sustainability [17] or reducing income inequalities [11] in healthy food consumption.

In this work, we focus on opinion formation models describing how opinions of individuals, also called agents or social actors, in an idealistic (time-invariant) closed community evolve in time under social influence. Reviews of common models of opinion evolution can be found in previous studies [14,18]. Among these, the discrete-time French-DeGroot model [19]—along with its continuous-time counterpart, for example, the Abelson model [20,21]—stands out as one of the earliest and most renowned. This model, like many of its later extensions, is grounded on the principle that individuals’ opinion updates occur through some weighted average of the opinions held by their neighbors within the community. Under specific assumptions on the connectivity of the network describing this cooperative community, the French-DeGroot model exhibits consensus, or, equivalently, it converges toward unanimous agreement on opinions [14]. Stemming from this fundamental model, there is now a rich literature on opinion-dynamics models incorporating cognitive features such as prejudices or stubbornness [22], exploring antagonistic compared with cooperative relations among individuals [23,24], and overall capturing more complex behaviors, including disagreement, clustering, and polarization.

This modeling approach based on opinion formation holds critical potential for studying the long-term impact of various socially determined behaviors, notably eating behaviors, which are significantly influenced by social factors and where decisions develop through close interaction with others [25]. The potential benefits of applying an opinion-dynamics approach, although based on a theoretical framework, include estimating individual food consumption outcomes over time despite data gaps, studying environmental impacts under different influence scenarios, and exploring how to design or modify an intervention [26]. It thus presents an opportunity to gain unique insights into how different policies could impact people’s dietary behaviors to the benefit of both human and planetary health.

Through the exploration of various scenarios involving social influence and policy implementation aimed at transforming dietary behaviors within a community using an integrated opinion formation model, this study’s overall aim was to provide novel insights into potential dietary shifts within the UK population. Specifically, this article leverages this theoretical modeling framework by grounding it in empirical data representing baseline UK dietary patterns and sociodemographic attributes, thereby providing a data-driven simulation of real-world policy implications. Following the consumption targets proposed by the UK Climate Change Committee (CCC) [27], these shifts are envisioned to be environmentally friendly, without disregarding social norms present in the community.

## Methods

### Data sources

To set up a food consumption baseline for the simulations, we used dietary data derived from the National Diet and Nutrition Survey (NDNS) 2019 [28]. The NDNS is a rolling program and provides information on people’s self-reported food intake over 4 d of a nationally representative, area-stratified, random sample. These data were chosen as they presently constitute the only nationally representative dietary intake data for the UK population. This survey contains consumption data from a nationally representative sample of 3558 individuals in the United Kingdom and information on each survey participant’s ID, age, sex (male/female), and socioeconomic status (high, medium, and low). The NDNS data provide quantities (in grams) of items eaten or drunk over 4 consecutive days, per main food group (e.g., fruit), subfood group (e.g., bananas), and per individual (discrete) food item (e.g., bananas raw) [28]. There are 60 main food groups, 147 subfood groups, and 3431 individual food items. For this analysis, we focused on changes in consumption of meat (red/processed and poultry meat), meat alternatives (e.g., tofu, tempeh, and Quorn), pulses (dry peas, beans, lentils, and chickpeas), and vegetables (fresh, canned, or frozen vegetables, excluding potatoes). These 4 food groups were selected as the most realistic substitutes for meat following methods from a previous work [4]. To estimate baseline intakes of these 4 food groups, we used 4 NDNS subfood groups for pulses, 1 subfood group for meat alternatives, 7 subfood groups for vegetables, and 10 main food groups for red, processed, and poultry meat (Supplemental Table 1).

Environmental impacts of meat, meat alternatives, pulses, and vegetables were based on data from a meta-analysis of food product life cycle assessments compiled from published literature related to UK-specific emission data [29] (Supplemental Table 2). These data were used to quantify the changes in diet-related greenhouse gas emissions (GHGEs), an estimate of the climate change impacts associated with each food product measured in kilograms of carbon dioxide equivalents (CO_2_eq); water use, measured in liters of blue water; scarcity-weighted water (weighted blue water use to produce food products by regional water availability) measured in liters; and land use (an estimate of how much arable land and pastureland is occupied to produce food product without biodiversity impacts), measured in square meters. The environmental impact data covers impacts for 57,185 unique food products sold in 8 UK-based and Ireland-based retailers. The impacts for individual foods are averaged for over 3000 retail categories (by department, aisle, and shelf), and are calculated per 100 g of food product.

**FIGURE 1:**
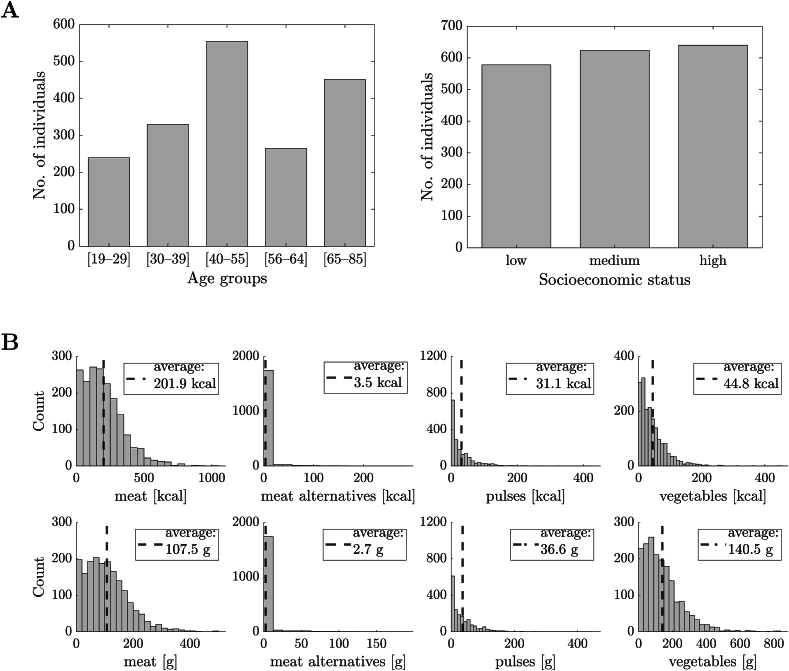
Histogram of the data obtained from the National Diet and Nutrition Survey 2019 in the United Kingdom [28]. (A) Sociodemographic attributes. Age groups (left panel) and socioeconomic status groups (right panel). (B) Baseline or initial conditions on consumption: recorded consumption of 4 food groups, i.e., meat, meat alternatives, pulses, and vegetables, in kilocalories (top panels) and grams (bottom panels). A vertical dotted line indicates mean values, also reported in the legend of each plot.

We considered the above-described survey data , excluding individuals *1*) for whom information regarding socioeconomic status is missing and *2*) whose age was <19 y. We obtained a sample of 1 841 individuals, divided into 5 different age groups (i.e., 19–29, 30–39, 40–55, 56–64, and 65–85 y) to map subcommunities sharing overlapping interests and 3 different socioeconomic status groups (i.e., low income, medium income, and high income). A summary of the data statistics (age groups, socioeconomic status, recorded consumption in kilocalories and grams) is given in Figure 1 [28].

### Targets

The sustainability aim explored in this article is aligned with the consumption targets for meat set by the UK CCC [27]. The CCC’s recommended Widespread Engagement Pathway represents a transition to net zero across all sectors of the economy, which involves ambitious behavioral change from consumers. The target is to achieve a specific percentage reduction in the average consumption of all meat and dairy products by 2050. However, in this case study, we only focus on changes in meat consumption and exclude dairy. The goal we address specifies 2 desired percentage decreases in the average consumption of meat, of 35% and 50%, to be achieved by the UK population on average by 2030 and 2050, respectively. These reductions are measured against the chosen baseline year 2019, when the NDNS survey was conducted [28]. Given the average initial meat consumption of ∼202 kcal/d (Figure 1B, top left panel), these targets translate into a desired maximum consumption of 131 kcal/70 g associated with the −35% target, and of 101 kcal/54 g associated with the −50% target.

### Opinion-dynamics modeling of food consumption

In this work, we simulate numerically and observe the opinion dynamics representing the time evolution of the opinions (i.e., dietary preferences) of a community of individuals connected in a social network, which models the UK population. We add to our modeling framework a virtual agent, representing external influence like that of governments or policymakers, connected to all agents in the UK population network. Consequently, we frame dietary shifts in the UK population as a problem of opinion formation with external influence. We observe this process over a sequence of campaigns, aimed at influencing opinions effectively and achieving convergence to a desired target (see Targets section), determined by an external entity.

Our simulations are built on the Friedkin-Johnsen (FJ) model (described in detail further). Simply put, this opinion-dynamics model is based on factors empirically shown to determine changes in people’s opinions when connected in a social network [14,22,30]. Essentially, there are 2 main sources of influence on people’s behaviors within a network: social influence (i.e., influence that occurs between peers connected in the social network) and external influence (i.e., influence that is exerted from an exogenous variable, e.g., a government). Changes in preferences/opinions and hence behaviors of individuals (or agents) in a network are captured and depend on *1*) each agent’s attachment to their initial opinions (in this case their baseline consumption meat), that is, their inertia toward diverging from their initial opinions; *2*) an influence factor that is represented by each agent’s susceptibility to their peers (i.e., the willingness/unwillingness to give up their own opinions in order to be more similar to their peers); and *3*) their susceptibility to external influence (such as a government) compared with that to initial opinions.

### Creation of the social network

Social psychological theory and social influence network theory suggest that human behavior is determined by the individual’s intention, which is influenced by their attitudes and by external influence stemming from their social network [[31], [32], [33]]. Eating behaviors can thus be seen as highly influenced by social factors, given that attitudes and habits develop in close interaction with other people. To study changes in dietary habits and assess potential health and environmental impacts, we, therefore, first set up the social network within which behaviors will be influenced and change. Further, we assume that each individual within the community, representing the UK population, is interconnected through a cooperative social network. This network is modeled as a weighted undirected graph of interactions, where the edge weights are determined by socioeconomic status (related to income) and age. In the opinion formation (described further), this means that the closer an individual is in age and socioeconomic status to their peers in the social network, the greater the influence those peers can exert over their opinion, and vice versa (Figure 1; Supplemental Information). These attributes were used for the formation of the network as they are thought to constitute environmental influences on food choices [34].

### Defining food opinions

The second and very central step in setting up the proposed opinion dynamics involves assigning each individual in the community with an initial meat opinion that represents their desired proportion of meat consumption in relation to their energy intake from meat and substitutes, indicated by a real number between 0 and 1 (Supplemental Figure 1). The energy intake from meat and substitutes is defined as the sum of the 4 abovementioned food groups: meat, meat alternatives, pulses, and vegetables. We assume that the changes in consumption of the remaining 3 food groups depend on the change in meat consumption, as well as each agent’s baseline consumption of meat alternatives, pulses, and vegetables, that is, their initial opinions regarding meat alternatives, pulses, and vegetables (Figure 1B). This dependency between meat and its potential substitutes (e.g., meat alternatives, pulses, and vegetables) is based on previous research [4,35,36]. However, although the health and environmental benefits of reducing animal product intake while increasing the consumption of plant-based foods in high-income countries are demonstrated [[4], [5], [6]], evidence from intervention and modeling studies concerning what happens to dietary patterns when meat consumption is reduced remains limited. Nevertheless, recent analyses of food consumption trends in the United Kingdom show that intakes of animal-source foods have declined whereas intakes of vegetables, in particular, have increased over the last 40 y [35]. Similarly, although the global average per capita consumption of meat is rising, livestock-derived food demand in some high-income countries is static or declining [36]. Furthermore, modeling studies and price elasticity calculations indicate that vegetables/legumes/meat alternatives are plausible substitutes for meat [37,38]. The market of the latter is projected to increase markedly over the coming 10–15 y [39], thus additionally supporting the substitution dependencies set out in our simulations.

In the opinion dynamics, we assume that it is unlikely that individuals consuming 0 meat will start consuming meat if meat-reducing policies are implemented. Similarly, (the few) individuals who currently do not consume any plant-based protein/vegetables are not likely to introduce much of it despite social influence. Hence, the model was set up so that vegetarians, that is, individuals whose percentage of meat consumption relative to their intake of meat and substitutes is 0% (number of vegetarians = 129), are fully stubborn with regard to both social and external influence. Hence, regardless of the influence of their peers and the external entity, they will remain anchored to their dietary habits. Carnivores, that is, individuals whose percentage of meat consumption relative to their energy intake is 100% (number of carnivores = 31) are fully stubborn with regards to social influence, but still susceptible to external influence (e.g., increased prices in meat following a meat tax might still have some impact on opinions/behaviors of meat eaters since that would affect financial prerequisites). Omnivores, that is, individuals whose percentage of meat consumption relative to their energy intake is strictly between 0% and 100%, can be partially stubborn with respect to their peers.

### Time evolution of opinions as per the FJ model

The time evolution of the opinions on meat consumption is described by a continuous-time counterpart of the FJ model [40] (Supplemental Information). The FJ model [22] is a well-known extension of the French-DeGroot model that accounts for partially or fully stubborn individuals, that is, agents that are partially or fully anchored to their prejudices, which are usually represented by their initial opinions but may also incorporate opinions from external sources of influence. The FJ model consists of 2 terms. The first term can be interpreted as a social cost and drives the agents toward consensus—like in the French-DeGroot model [19]. The second term can be interpreted as an inertia cost and reflects the agents’ reluctance to deviate from their prejudices [41]. For each agent, the trade-off between the 2 terms is governed by their susceptibility, denoted for each agent i by $\lambda_{i}$ in Equation S3 in Supplemental Information. We say that an individual i is fully stubborn if $\lambda_{i}=0$ (such as vegetarians or carnivores), and partially stubborn if $\lambda_{i}\in \left(0,1\right)$ (such as omnivores). Typically, this model converges to disagreement (i.e., consensus is not achieved): a feature of this model is that the final opinion of any agent, that is, intuitively the opinion reached after a significant amount of time has passed, is a convex combination of prejudices. The choice of the FJ model, aside from theoretical attractiveness (i.e., it is a linear model with known dynamical properties), is inspired by the work of Friedkin et al. [42], where the authors use a (multidimensional) FJ model to describe the evolution of the distribution of food-related preferences of a group of individuals and report findings from resource allocation experiments. More details on how the FJ model was applied in our simulations can be found in the Supplemental Information.

### External influence

To include an external influence on the agents’ behaviors, an additional virtual node, that is, an influencer agent or external entity, is linked to all agents in the food network. To capture the influence of the external entity on the dynamics of opinion formation, we model each individual’s prejudice as a trade-off between their initial opinion and the opinion of the external entity (Equation S4 in Supplemental Information). We use the parameter $\gamma_{i}\in \left[0,\bar{\gamma }\right]$ to model the influence efforts of the external entity toward each agent i. When $\gamma_{i}=0$, the external entity has no influence on the agent i, that is, in the absence of external influence, initial opinions (interpreted as dietary habits) are the only source of prejudice (i.e., inertia). The maximum value $\bar{\gamma }$ represents the maximum extent of influence that the agents are willing to accept or can tolerate. Finally, given the context of dietary shifts, it is anticipated that a certain amount of time is required before any noticeable change occurs within the UK population. For this reason, we promote the idea that the external entity “acts,” changing or updating the prejudices of the individuals in the population, only when the observed behavior of the social network is stationary. For our modeling approach, we use a modified concatenated FJ model [43] over a sequence of campaigns (Supplemental Information).

In this work, we consider 2 different settings for the external influence parameters $\gamma_{i}$, i,…,n, where n is the total number of individuals in the UK population under investigation, the first being our main simulation setting and the second representing a sensitivity analysis. To compare the different settings, we compute the time-to-target associated with the −35% and −50% targets for decreased meat consumption. A third setting of external influence under budget constraints is presented in the Supplemental Information [40].

### High compared with low external influence

The main simulation setting is that of a so-called broadcast external influence, or equal influence, among the agents, that is, $\gamma_{i}=\bar{\gamma }$ for all i=1,..,n. We select 2 values of $\bar{\gamma }$, that is, $\bar{\gamma }=0.025$ and $\bar{\gamma }=0.025/2=0.0125$, capturing high and low influence, respectively. High influence represents a scenario where policymakers implement nationwide information campaigns for reduced meat consumption in conjunction with fiscal measures (taxes on meat + subsidies on meat alternatives/pulses/vegetables). This approach builds on previous research suggesting that a multifaceted policy package would be the most appropriate to achieve shifts toward sustainable (low-meat) diets [44]. In contrast, low influence represents a scenario where policymakers implement a nationwide information campaign for reduced meat consumption only—information campaigns/dietary guidelines have been shown to have limited impact on changing people’s diets [44].

### Sensitivity analysis

#### Varied external influence

As an alternative simulation setting, we modified the external influence so that it was not exerted equally among agents. Instead, we explored the effects of targeting the population based on their baseline food opinions. We did this as a sensitivity analysis, to compare different approaches with external influence (i.e., the effectiveness of universal compared with more individual-based policies). Thus, we applied diverse values of influence between the individuals, that is, $\gamma_{i}\neq \gamma_{j}$for all i,j=1,…,n. We considered 2 cases. The first was used to test heterogeneous external influence parameters, generated as uniformly or normally distributed random numbers, whereas the second introduced a dependency between the baseline percentage of meat consumption and the influence parameters.

First, we uses a uniform distribution centered in 0.0125 (i.e., the value adopted in the low-influence scenario in the broadcast setting)—$\gamma_{i}∼U_{\left[0,0.025\right]}$ for all i, from which we drew 500 samples, obtaining in median an approximate time-to-target of 10.1 y. In addition, to assess the impact of skewed distributions, we considered a normal distribution truncated between 0 and 0.025 with a SD equal to σ=0.2×0.025 and varying mean μ∈{0.025×0.05,0.025×0.1,..,0.025×0.95}, that is, $\gamma_{i}∼N_{\left[0,0.025\right]}\left(\mu ,\sigma \right)$ (Supplemental Figure 2A, left panel). For small values of μ, the distribution becomes more concentrated near 0, whereas for higher values of μ, it becomes more spread out toward 0.025. For each value of μ, we drew 500 samples from the corresponding distribution and computed the time needed to reach the −35% target (Supplemental Figure 2A, right panel).

Second, to correlate external influence and meat consumption, we considered the above-described truncated normal distribution, but we sorted the external influence parameters according to the percentage of meat consumption at baseline (Supplemental Figure 2B, left panels): this was captured by the ordering o:{1,…,n}↦{1,…,n} such that $x_{o\left(1\right)}\left(0\right)\leq \ldots \leq x_{o\left(n\right)}\left(0\right)$, where $x_{i}\left(0\right)$ indicating the initial opinion of the agent i. This yields $\gamma_{o\left(i\right)}∼\text{sorted}{\left(N_{\left[0,0.025\right]}\left(\mu ,\sigma \right)\right)}_{i}$. The aim was to assess the time-to-target by considering both the cases in which the external entity had a stronger influence on individuals with a smaller proportion of meat consumption (Supplemental Figure 2B, bottom panels) and where the external entity predominantly influenced individuals with a higher proportion of meat in their diet (Supplemental Figure 2B, top panels).

#### Varied conditions for allocation of consumption across food groups

We tested 2 different approaches with regards to the conditions for how individuals allocate energy intake across meat alternatives, pulses, and vegetables following a decrease in meat consumption. Our main approach followed the previously described assumption that the changes in consumption of the remaining 3 food groups depend on the change in meat consumption, as well as each agent’s baseline consumption of meat alternatives, pulses, and vegetables, that is, their initial opinions regarding meat alternatives, pulses, and vegetables. For the 436 individuals consuming only meat or only meat and vegetables, but no meat alternatives or pulses, at baseline, we introduced some flexibility so that they are allowed to consume some amount of meat alternatives and pulses when meat consumption reduced. We did this mainly to account for the uncertainty in our dietary data; there was a chance that meat alternatives and pulses might not have been reported during the 4-d diary days but still be food that would fit into a person’s weekly or monthly diet. Our alternative approach was to follow the assumption that the changes in consumption of the remaining 3 food groups depend strictly on each agent’s baseline consumption of meat alternatives, pulses, and vegetables (Supplemental Table 3).

#### External influence under budget constraints

In the last simulation, detailed in the Supplemental Information as it was not the primary focus of this analysis, we examined the impact of budget constraints on campaigns organized over a 5.2-y span (i.e., the time-to-target in the high influence broadcast scenario) (Supplemental Figure 3). Specifically, we explored 2 situations as follows: 1) the external entity was able to influence all agents in each campaign, but a budget constraint limited the total number of campaigns where this influence could occur; and 2) for each campaign, a budget constraint limited the total number of agents that could be influenced.

### Metrics of evaluation

Our focus lied in assessing various metrics relative to the targets established by the UK CCC. These metrics included the average and individual relative changes in meat consumption (scaled) calculated at each time point in comparison with the baseline (Equation S4 in Supplemental Information), the time-to-target, defined as the minimum number of weeks (noted at the campaigns’ end) required to reach the −35% and −50% targets (Equation S5 in Supplemental Information), and the number of adopters, defined for each time as the number of individuals in the UK population who consume less than the desired target for meat (Equation S6 in Supplemental Information). Finally, we evaluated the absolute and relative changes in consumption of meat, meat alternatives, pulses, and vegetables, as well as changes in environmental impacts GHGE, water use, scarcity-weighted water, and land use (Supplemental Table 2).

## Results

### Baseline consumption

The distributions of the baseline consumption of meat, meat alternatives, pulses, and vegetables are shown in Figure 1. Data from the NDNS 2019 [28] (Supplemental Table 1) provided the initial conditions of our analysis and showed that the mean daily intake of meat is 129.9 and 91.6 g for males and females, respectively (Table 1). Mean daily intakes of meat alternatives were low, with males having a mean daily intake of 2.6 g and females a mean daily intake of 2.7 g. Mean daily intakes of pulses were also relatively low at 41.7 and 33.0 g for males and females, respectively. As for vegetables, the mean daily intake was 135.6 g for males and 143.9 g for females. At baseline consumption, the share of meat relative to the total amount of meat plus substitutes was >75% in 50% of the surveyed UK population (Supplemental Figure 1, leftmost panel).

**TABLE 1:** Absolute change between baseline consumption and numerical simulation at the time-to-targets $t_{35\%}=270$ and $t_{50\%}=420$ wk (Equation *S6* in Supplemental Information) associated with desired percentage decreases equal to −35% and −50%, respectively, under a high-influence scenario. A:. B:.

Consumption (g)	Males		Females	
	mean	SD	mean	SD
Daily per capita consumption of food groups in grams				
Meat				
Baseline consumption	129.9	84.5	91.6	64.9
At week 270 (∼5.2 y)	83.9	49.5	62.0	39.3
At week 420 (∼8.1 y)	63.7	37.2	47.4	29.7
Meat alternatives				
Baseline	2.6	12.3	2.7	13.6
At week 270 (∼5.2 y)	3.7	13.6	3.4	13.8
At week 420 (∼ 8.1 y)	4.1	14.7	3.8	14.7
Pulses				
Baseline consumption	41.7	51.5	33.0	39.3
At week 270 (∼5.2 y)	101.0	84.5	68.5	53.0
At week 420 (∼8.1 y)	124.7	105.7	83.1	64.6
Vegetables				
Baseline	135.6	110.5	143.9	113.7
At week 270 (∼5.2 y)	304.5	229.6	258.7	173.7
At week 420 (∼8.1 y)	378.3	295.4	315.7	219.2
Environmental impacts from Supplemental Table 2 (daily per capita impacts from meat, meat alternatives, pulses, and vegetables, respectively)				
kgCO_2_eq				
Baseline consumption	3.2	1.9	2.3	1.5
At week 270 (∼5.2 y)	2.4	1.3	1.8	1.0
At week 420 (∼8.1 y)	2.1	1.1	1.6	0.9
m^2^_LandUse_				
Baseline	6.9	4.1	5.0	3.1
At week 270 (∼5.2 y)	5.5	3.0	4.1	2.3
At week 420 (∼8.1 y)	4.9	2.7	3.6	2.0
L_WaterUse_				
Baseline	203.0	107.1	156.2	82.5
At week 270 (∼5.2 y)	201.4	108.5	156.2	83.3
At week 420 (∼8.1 y)	200.0	112.1	155.5	85.1
L_Scarcity-weighted water_				
Baseline	5400.6	3071.9	3932.5	2331.0
At week 270 (∼5.2 y)	4626.6	2494.3	3416.6	1857.6
At week 420 (∼8.1 y)	4260.8	2320.1	3129.2	1682.4

### Changes in food consumption under different broadcast scenarios

**FIGURE 2:**
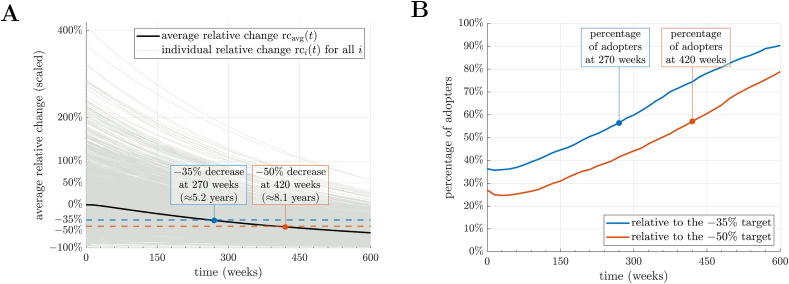
Performance indicators during repeated campaigns to reduce the consumption of meat, with decrease targets equal to −35% and −50% (high-influence scenario). (A) Scaled average relative change (Equation S4a in Supplemental Information), indicated by a thick black line, and individual relative changes across the population (Equation S4b in Supplemental Information), represented by light gray lines. Dashed lines indicate the desired decrease in meat consumption and the 2 circles the corresponding time-to-target (Equation S5 in Supplemental Information). (B) Percentage of adopters (Equation S6 in Supplemental Information) relative to the −35% (blue line) and −50% (red line) targets. Observe that the percentage of adopters at the end of the campaign is <100% since not all agents achieve the desired (global) target on meat consumption (dkcal; see Methods).

**FIGURE 3:**
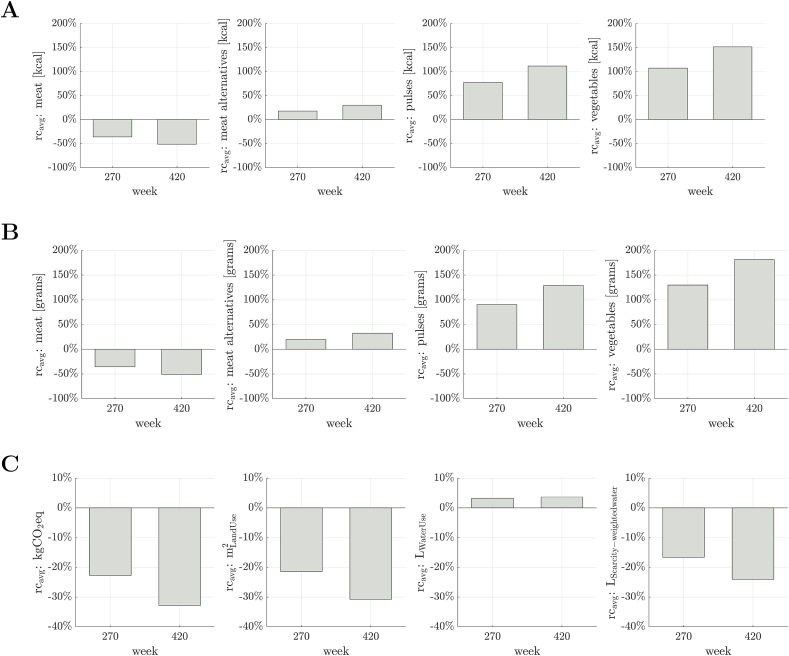
Average relative change at time-to-targets (Equation S6 in Supplemental Information) corresponding to 35% and 50% targets decrease in meat consumption, respectively (high-influence scenario). (A) For the consumption of all food groups in kcal. (B) For the consumption of all food groups in grams. (C) For the environmental impacts from Supplemental Table 2.

Our simulations showed that, under a high governmental influence scenario, the −35% target for meat consumption in the UK population would be met at 270 wk (∼5.2 y or well within the timeframe suggested by the CCC) (Table 1; Figure 2A) with the percentage of adopters being 56.4% (Figure 2B). In absolute terms, reaching the –35% target would correspond to an average daily reduction of −46 and −29.6 g of meat for males and females, respectively (Table 1). For the population as a whole, achieving this target would lead to a 34.3% grams increase in meat alternatives, a 129.7% increase in pulses, and a 102.8% increase in vegetables (Figure 3B). As for environmental impacts (Supplemental Table 2), we observed a 23.8% decrease in GHGE, a 20.2% decrease in land use, a 0.5% decrease in water use, and a 14.5% decrease in scarcity-weighted water (Figure 3C).

Reaching the −50% consumption target for meat under the high-influence scenario would take 420 wk (∼8.1 y, again within the CCC target) (Table 1; Figure 2A), with the percentage of adopters being 57.1% (Figure 2B). In absolute terms, reaching the −50% target would correspond to an average daily reduction of −66.2 and −44.2 g of meat for males and females, respectively (Table 1). In parallel with reductions in meat consumption, the grams intake of meat alternatives, pulses, and vegetables would increase by 49.1%, 178.4%, and 147.0%, respectively, for the population as a whole (Figure 3B). This translates into a 34.1% decrease in GHGE (CO_2_eq), a 29.3% decrease in land use, a 1.0% decrease in water use, and a 21.4% decrease in scarcity-weighted water (Figure 3C).

**FIGURE 4:**
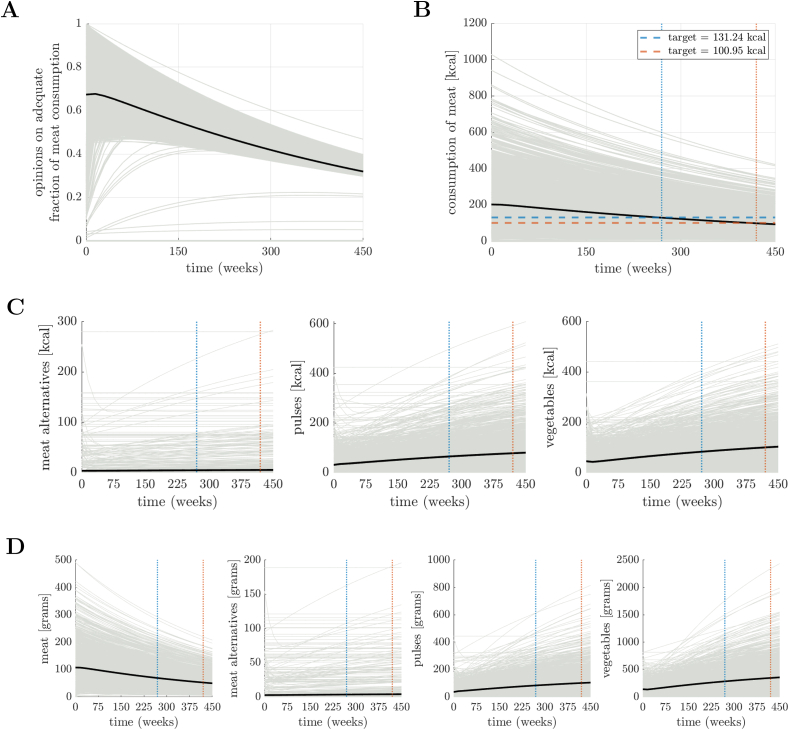
Repeated campaigns to reduce the consumption of meat (Equation S3 in Supplemental Information) (high-influence scenario). (A) Opinions of agents regarding fraction consumption of meat in their diet with respect to their energy intake from meat and substitutes. (B) Corresponding consumption of meat in kcal. Observe the presence of vegetarians in (A) and (B), indicated by a flat line at zero. A dashed line indicates the desired target for meat consumption corresponding to a −35% (blue color) and −50% (red color) decrease. (C) Consumption of all food groups in kcal. (D) Consumption of all food groups in grams. In all panels, a black line indicates the average (opinion or consumption), whereas vertical dotted lines indicate the time-to-target (Equation S6 in Supplemental Information).

The opinion formation on meat consumption and the corresponding time evolution of meat consumption in kilocalories for the high-influence scenarios are shown in Figure 4A and B, respectively. The time evolution of all food group consumption in kilocalories and grams is given in Figure 4C and D, respectively. Note that vegetarians (i.e., individuals with zero meat consumption at baseline) maintained their dietary habits throughout the simulation period and thus did not change their consumption in any food group, that is, meat alternatives, pulses, and vegetables (e.g., constant lines in Figure 4B). Correspondingly, the time evolution of the environmental impacts across all 4 food groups is illustrated in Supplemental Figure 4.

Under a low governmental influence scenario, a longer time was needed to meet the UK CCC’s consumption targets, and we observed a delay of 225 wk for the −35% target and 345 wk for the −50% target. Indeed, although the −35% target was met at 270 wk (∼5.2 y) in the case of high influence (Figure 2A), it was not achieved until 495 wk (∼9.5 y, which would be outside the timeframe outlined by the CCC for changes implemented in 2024) in the low-influence scenario (Supplemental Figure 5A). Similarly, although the −50% target was met at 420 wk (∼8.1 y) in the case of high influence (Figure 2A), it was not achieved until 765 wk (∼14.7 y) in the low-influence scenario (Supplemental Figure 5A). This would still be achieved before the 2050 target set by the CCC if changes were implemented in 2024. Despite the delay, the changes to food consumption and environmental impacts at 495 and 765 wk were similar to the ones obtained in the high-influence scenarios (Supplemental Figure 6).

### Sensitivity analysis—modifying external influence and conditions for allocation of consumption across food groups

In comparison with the above-described broadcast scenarios, the first part of our sensitivity analysis proposed simulations with diverse individual values of external influence. The first approach showed that, when influence values are uniformly assigned, ≥10 y are needed to meet the UK CCC’s −35% target for meat consumption (details in Supplemental Information 2.3.2), not far from the 9.5 y corresponding to the low uniform influence scenario. When values are assigned according to a truncated normal distribution with a varying mean (Supplemental Figure 2A, left panel), the time required to reach the target ranges from 6 to 20 y (Supplemental Figure 2A, right panel). The shorter duration corresponds to a distribution concentrated near the maximum influence (high influence overall), whereas the longer duration corresponds to a distribution concentrated near 0 (small influence overall). In the second approach, where influence values were assigned according to a truncated normal distribution and correlated with the baseline distribution of meat consumption/meat opinions (Supplemental Figure 2B, left panels), the time required to achieve the 35% reduction target in meat consumption varies significantly, from ∼5 to 30 y (Supplemental Figure 2B, right panels). This variation depended on which group was predominantly influenced by the external entity: individuals with a higher proportion of meat consumption at baseline in the first case and individuals with a smaller proportion of meat consumption in their baseline diet in the second case.

Changing the conditions for how individuals allocate their consumption across meat alternatives, pulses, and vegetables following a decrease in meat consumption did not result in any major discrepancies compared with the results presented. However, we observed a slight increase in water use from an illustrative analysis that heavily favored the consumption of vegetables (Supplemental Table 3).

## Discussion

The results from our opinion-dynamics simulations demonstrate how various scenarios integrating social influence between peers and external policy implementation could reshape dietary choices at a population level in the United Kingdom over the coming decades. Reaching CCC’s 35% and a 50% reduction in meat consumption under high policy influence in the UK population could take between ∼5 and 10 y, respectively, well within the timeframe specified by the CCC (with changes implemented in 2024). Achieving these consumption targets in the high-influence simulation would also lead to noticeable increases in the consumption of meat alternatives, pulses, and vegetables, as well as important reductions in GHGE, land use, water use, and scarcity-weighted water use. A lower external influence would increase the time-to-target to ∼10 and 15 y for the same consumption targets and would lead to similar changes in food consumption and environmental impacts.

### Comparison with other studies of meat consumption in the United Kingdom

Similar to previous simulations of meat consumption in the United Kingdom [15], our work showed that a high external influence (information campaigns/fiscal measures) was more efficient in reducing meat consumption over time than a lower external influence (information/social marketing campaigns). Furthermore, our high-influence scenario showed a slightly greater reduction in meat consumption at 3 y than a previous simulation [15] testing the impact of a tax on red and processed meat (−23% compared with −21% reduction). The slight difference can be explained by differences in modeling approaches, including the fact that our high influence approach represented a scenario, which was modeled to represent both fiscal measures and information campaigns.

In the sensitivity analyses, we explore the effects of different approaches to targeting the population based on their baseline food opinions as opposed to implementing a broadcast (universal) external influence. This resulted in slower progress toward the CCC’s targets, especially in the case (taking almost 25 y) where the external entity exerted a stronger influence on individuals with a low meat consumption at baseline. In this case, the external entity directs all its influence efforts toward agents who already hold a favorable opinion toward the desired target, whereas disregarding those who are distant from it. Consequently, these individuals may have small motivation to change or may be unable to achieve any change at all, thus slowing down the evolution of the network’s opinions over time. This tallies findings from previous simulations [15] that observed boomerang effects (increased rather than reduced meat consumption among agents) when only agents who were the most concerned about eating sustainably were targeted by social marketing campaigns aimed at reducing consumption. In contrast, the case where the external entity exerted a stronger influence on individuals with a high meat consumption at baseline, was much more effective than the opposite case, with a time-to-target slightly longer than that of the high-influence broadcast scenario. This is likely to be a less realistic option when involving food taxes and subsidies in practice, since such measures are universal by nature. The dietary data indicate that, at baseline, males consume more meat than women, which is a well-known pattern in dietary habits. In the broadcast scenario, where no difference is imposed between individuals regarding external influence, simulations show that the 35% target translates into an average decrease of −46 g for males and −29.6 g for females (see Table 1). In practice, a greater effort (in terms of altering food habits) is likely to be required from males, and so, outside of a simulation environment, they might need to be targeted more intensely/differently than females. This highlights the importance of better understanding gender-based differences and drivers in the process of reducing meat consumption [45], as well as ways of accounting for that complexity (e.g., by incorporating targeted influence alongside universal influence) in future ABMs.

### Strengths and limitations

To the best of our knowledge, this study presents a novel approach to test opinion dynamics in the context of social and external influence on food consumption, providing a novel way of understanding the evolution of dietary behaviors. An important strength of our modeling approach is that it implements and assesses the behavioral effects of both social and external influence. Compared with looking at social and external pathways in isolation, these simulations are therefore likely to capture a more complete picture of the evolution of food opinions among a large group of connected individuals that are part of a complex (food) system. Our study also explores different scenarios for external (governmental) influence, thus providing policymakers with some indication of plausible efforts needed to reach governmental goals along with timelines to be expected from such efforts [[46], [47], [48]].

An insight emerging from this research is the intuitive observation that, in general, reaching the −35% or −50% targets on meat consumption might not guarantee a decrease in the projected environmental impacts. For example, our sensitivity analysis shows that by changing the conditions for consumption and removing some degree of flexibility in the opinion formation, we observed slight increases (2.5% and 3.7% for males and females, respectively) (Supplemental Table 3) in water use in correspondence to the −35% target in meat consumption (at 270 wk), in contrast to the decreases obtained in the balanced case illustrated in Table 1. The increase in water use results from a slightly higher increase in vegetables and pulses (as compared with the increase resulting from our initial consumption rules), which are food groups that have a relatively high demand for freshwater [49]. However, including some flexibility with regard to the consumption of meat alternatives and pulses for some agents did not result in increases in any environmental impacts. Although the latter approach is possibly a more balanced design choice, it might still be too simplistic compared with a reality that is likely to be more unpredictable and complex than our modeled context. Hence, the consumption shifts under our modeled scenarios could, in practice, lead to a different allocation of meat alternatives, pulses, and vegetables with implications for the environment.

This approach is constrained by the theoretical framework we have adopted, which limits the (empirical) scope of the analysis, and future directions include the collection and analysis of longitudinal data. Our simulations are also limited by only considering changes in the consumption of 4 food groups. Although our choices of substitute foods were supported by previous research [29], [[49], [50], [51]], dietary substitutions could possibly occur across a broader range of food groups. Hence, future simulations should aim at looking at changes in diets as a whole. Furthermore, our 2 different influence scenarios (high and low) lack detail in the sense that we only hypothesized that a high external influence would represent fiscal measures and information without incorporating information price elasticities as done previously by Scalco et al. [15]. Our assumptions regarding parameter settings in the opinion dynamics, however, seem to have been fairly appropriate since our results closely mirror those presented by Scalco et al. Finally, the survey data used as initial conditions in this work are from 2019 [28]; therefore, the dietary data may not accurately represent current diets. However, since we are modeling relative rather than absolute reductions in consumption, this may not have materially affected the estimates.

Applying our theoretical modeling framework to nationally representative consumption data and insights from published research, our analyses bring together nationally representative consumption data and published research to explore potential joint benefits for people’s dietary intakes (relative to national goals) and the environment. However, our simulations are limited in the way that they do not account for potential trade-offs concerning other aspects of sustainability such as nutrition or health. There is a risk that the dietary changes observed lead to some nutritional imbalances, such as deficiencies in iron, zinc, and vitamin B-12, which are more densely available and more bioavailable in meat than plant-protein sources and vegetables [50]. However, previous health impact models on dietary shifts [4,6,51] support the idea that the consumption shifts observed in our simulations are likely to accrue substantial population health benefits.

## Conclusions

By investigating the evolution of dietary behaviors using an empirically founded opinion-dynamics model, incorporating social influence between peers, and through a range of different plausible scenarios for external influence, our work adds new evidence to support the introduction of policies to meet meat reduction targets in the United Kingdom. The presented estimated benefits for consumption and the environment carry significant uncertainty and are likely to not provide a full representation of reality. Nevertheless, they should help determine the order of magnitude of positive outcomes that might be expected and thus lend important weight to support policymakers in their review of options to implement meat reduction.

## Author contributions

The authors’ responsibilities were as follows – AF, PEC: designed and conducted research and analyzed the data; all authors: discussed the results and wrote the paper; AF, PEC: were responsible for the final content of the manuscript; and all authors: have read and approved the manuscript.

## Data availability

Data described in the manuscript, code book, and analytic code will be made publicly and freely available without restriction.

## Funding

This project received funding the Swedish Research Council (VR, grant nr. 2022-00344) for the submitted work. The Swedish Research Council had no role in the design, analysis or writing of this article.

## Conflict of interest

RG is an Editor for *Current Developments in Nutrition* and played no role in the Journal’s evaluation of the manuscript. All other authors report no conflicts of interest.
