# Supplementary material for: Social Interactions for Sustainable Food Choices: Meeting the Target for Meat Intake in the United Kingdom
Source: Curr Dev Nutr. 2025 Jul 30;9(9):107509. doi: 10.1016/j.cdnut.2025.107509 (PMC12512162; doi:10.1016/j.cdnut.2025.107509)
Supplement: Multimedia component 1 [file mmc1.pdf]

Supplementary Information for

Social interactions for sustainable food choices: Meeting the target for meat intake in the United Kingdom

Angela Fontan[1], Rosemary Green[2], Karl Henrik Johansson[1], Patricia Eustachio Colombo[2,3,*]

[1] Division of Decision and Control Systems, School of Electrical Engineering and Computer Science KTH Royal Institute of Technology, SE-100 44 Stockholm, Sweden. E-mail: angfon@kth.se. This author is also affiliated with Digital Futures, Stockholm, Sweden.

[2] Centre on Climate Change and Planetary Health, London School of Hygiene and Tropical Medicine, Keppel St, London WC1E 7HT, UK.

[3] Department of Biosciences and Nutrition, Karolinska Institutet, 17177 Stockholm, Sweden Department of Global Public Health. Karolinska Institutet, Stockholm, Sweden.

[*] Correspondence: Patricia Eustachio Colombo, patricia.eustachio-colombo@lshtm.ac.uk

Contents

1 Target on consumption of meat **2**

2 Opinion dynamics modeling food consumption **2**
- 2.1 The Friedkin-Johnsen model of opinion dynamics . . . 3
- 2.2 Parameters of the opinion forming model . . . 4
  - 2.2.1 Creation of the UK population “food” network . . . 4
  - 2.2.2 Vegetarians, carnivores, and omnivores . . . 5
- 2.3 External influence scenarios in the numerical simulations . . . 5
  - 2.3.1 Broadcasting - high vs. low influence . . . 6
  - 2.3.2 Sensitivity analysis . . . 6
  - 2.3.3 Budget constraints and targeted campaigns . . . 7

3 Metrics **8**

List of Figures

1 Distribution of baseline consumption . . . 10
2 Time-to-target for different distribution of γ_i . . . 11
3 Average relative change under budget constraints . . . 12
4 Environmental impacts . . . 13
5 Performance indicators, low influence . . . 14
6 Average relative change, low influence . . . 15

List of Tables

1 Simulated food groups and corresponding NDNS subfood groups . . . 16
2 Values for the environmental impacts per 100g of food . . . 16
3 Absolute changes, for a different choice of food groups proportions. . . . 17

1 Target on consumption of meat

We consider the UK Climate Change Committee's (CCC) consumption targets for meat [1], targets to be achieved on average in the population. The CCC's recommended Widespread Engagement Pathway represents a transition to net zero across all sectors of the economy which involves ambitious rates of behavioral change from consumers. The target is to achieve a specific percentage reduction in the average consumption of all meat and dairy products by 2050. In this case study, we only consider changes in meat consumption and exclude dairy. The desired percentage decreases in the average consumption of meat are 35% and 50%, which we denote by $d_{\%}$. Given $d_{\%} \in \{35\%, 50\%\}$, the target for meat consumption in kcal, denoted by $d$kcal, can be specified as $d\text{kcal} = (1 - d_{\%})\frac{1}{n}\sum_{i=1}^{n} x\text{kcal}_i(0)$. Here, $x\text{kcal}_i(0)$ represents the initial consumption of meat of agent i collected via the survey (the notation will be clarified in Supplementary Section 2), and $\frac{1}{n}\sum_{i=1}^{n} x\text{kcal}_i(0)$ represents the average consumption of meat in the population at baseline. Using the data collected in the survey (see Figure 1 in the main manuscript), we can observe that $\frac{1}{n}\sum_{i=1}^{n} x\text{kcal}_i(0) = 202$ kcal (approximately); thus $d\text{kcal} = 131.24$ kcal when $d_{\%} = 35\%$, and $d\text{kcal} = 100.95$ kcal when $d_{\%} = 50\%$.

2 Opinion dynamics modeling food consumption

In this work, agents' opinions in the population represent their beliefs regarding a balanced distribution among 4 food groups, i.e., meat, meat alternatives, pulses, and vegetables, of the energy intake from meat and substitutes. For simplicity, we posit that the agents discuss their preferences regarding meat consumption with their neighbors in the food network (details in Supplementary Section 2.1). For each agent, the distribution of the other food groups of interest (meat alternatives, pulses, and vegetables) is based on a fixed proportion with respect to the energy intake from substitutes, determined by the proportions observed in the recorded NDNS survey [2] (see Remark 1).

Remark 1 (Distribution of the food groups meat alternatives, pulses, and vegetables)**.** *For each agent $i = 1, \ldots, n$, where n denotes the total number of agents, let E_i denote the energy intake from meat and substitutes in kcal, and let the parameters $\alpha_{i,\text{meat alternatives}}$, $\alpha_{i,\text{pulses}}$, and $\alpha_{i,\text{vegetables}}$, denote fixed proportions of consumption of each food group with respect to the energy intake from substitutes. These fixed proportions are obtained from the survey as follows:*

$$\alpha_{i,\text{meat alternatives}} = \frac{\text{baseline consumption of meat alternatives in kcal}}{E_i - \text{baseline consumption of meat in kcal}}$$
$$\alpha_{i,\text{pulses}} = \frac{\text{baseline consumption of pulses in kcal}}{E_i - \text{baseline consumption of meat in kcal}}$$
$$\alpha_{i,\text{vegetables}} = \frac{\text{baseline consumption of vegetables in kcal}}{E_i - \text{baseline consumption of meat in kcal}}.$$

For these parameters it holds that $\alpha_{i,\text{meat alternatives}}$, $\alpha_{i,\text{pulses}}$, $\alpha_{i,\text{vegetables}} \in [0, 1]$ and $\alpha_{i,\text{meat alternatives}} + \alpha_{i,\text{pulses}} + \alpha_{i,\text{vegetables}} = 1$, where the second constraint ensures that the energy intake from meat and substitutes is constant for each agent. Accordingly, the consumption of meat alternatives, pulses, and vegetables in kcal for each agent $i = 1, \ldots, n$ at time t is given by:

$$\text{consumption in kcal of meat alternatives}_i(t) = \alpha_{i,\text{meat alternatives}}\,(E_i - x\text{kcal}_i(t))$$
$$\text{consumption in kcal of pulses}_i(t) = \alpha_{i,\text{pulses}}\,(E_i - x\text{kcal}_i(t))$$
$$\text{consumption in kcal of vegetables}_i(t) = \alpha_{i,\text{vegetables}}\,(E_i - x\text{kcal}_i(t))$$

where $x\text{kcal}_i(t)$ denotes the consumption of meat in kcal for agent i at time t, whose evolution in time is detailed in the next section.

The dynamical model considered in this manuscript describes changes in meat consumption due to social influence and external influence from sequential campaigns. In general, achieving the desired reduction in meat consumption may prove challenging with just one campaign. To address this issue and effectively influence behavior, we consider the case of repeated campaigns. The model is based on the works [3, 4] and [5], and consists of three key components: (1) A model for the evolution of opinions intra-campaign; (2) A rule for external influence to achieve the target meat consumption throughout the campaign sequence. (3) A rule for concatenating opinions between campaigns.

2.1 The Friedkin-Johnsen model of opinion dynamics

Let $E_i > 0$ be the (fixed) energy intake from meat and substitutes for each agent i, $i = 1, \ldots, n$, where n denotes the total number of agents. The opinion of agent i at a certain point in time t regarding their preferred fraction consumption of meat in their diet with respect to their energy intake from meat and substitutes is described by $x_i(t) \in [0, 1]$, i.e., $x_i(t) = x\mathrm{kcal}_i(t)/E_i$ for all i, where $x\mathrm{kcal}_i(t) \in \mathbb{R}^+$ ($x\mathrm{kcal}_i(t) \leq E_i$ for all t) denotes the consumption of meat in kcal by agent i. $x\mathrm{kcal}_i(0)$ represents the initial recorded consumption of the agent i obtained through the survey (expressed in kcal), and $x_i(0)$ the initial opinion expressed as a fraction w.r.t. E_i, i.e., $x_i(0) = x\mathrm{kcal}_i(0)/E_i$.

To capture social interactions within the population, we assume that the agents are connected by a graph $\mathcal{G} = (\mathcal{V}, \mathcal{E}, A)$, where $\mathcal{V} = \{1, \ldots, n\}$ is the set of nodes ($\mathrm{card}(\mathcal{V}) = n$ is the total number of agents in the population), $\mathcal{E}$ is set of edges, and $A = [a_{ij}] \in \mathbb{R}^{n \times n}$ is the adjacency matrix. In the context of social networks, each node of $\mathcal{G}$ represents an agent (note that the terms "agent" and "individual" will be used interchangeably in this work), and each element $a_{ij} \geq 0$ represents the amount of trust between agents j and i, with $a_{ij} > 0$ if and only if there exists an edge $(j, i) \in \mathcal{E}$ between j and i. In this work, we assume that the graph $\mathcal{G}$ is strongly connected, meaning that there exists a (directed) path–a sequence of directed edges–from any node $i \in \mathcal{V}$ to any other node $j \in \mathcal{V}$.

We posit that the evolution in time of the opinion of each agent $i \in \mathcal{V}$ regarding a balanced share of meat consumption with respect to the energy intake from meat and substitutes is described by the following continuous-time version of the Friedkin-Johnsen (FJ) model[1]:

$$\dot{x}_i(t) = \lambda_i \sum_{j=1}^{n} a_{ij}(x_j(t) - x_i(t)) + (1 - \lambda_i)(u_i - x_i(t)). \tag{1}$$

The parameter $\lambda_i \in [0, 1]$ represents individual's i *susceptibility* to interpersonal influence or, equivalently, $1 - \lambda_i$ indicates the level of *prejudice* of individual i, that is, the attachment to their own prejudice u_i: a low (resp., high) value of λ_i (resp., $1 - \lambda_i$) indicates a prejudiced (resp., susceptible) individual, that is, an individual that is less (resp., more) prone to change its opinion.

The convergence properties of the model (1) are well-known and are discussed in [8]. The key result is that, under the assumptions that the graph is strongly connected and there is at least one prejudiced agent (for which $\lambda_i < 1$), then the opinion of each agent converges in time to a fixed value, which is a convex combination of the prejudices of all the agents in the food network. Theoretically, it holds that $x(\infty) := \lim_{t \to \infty} x(t) = Vu$ where $x(t) = [x_1(t) \cdots x_n(t)]^T$ is the vector of opinions, $u = [u_1 \cdots u_n]^T$ is the vector of prejudices (typically, $u = x(0)$), and $V = (\Lambda L + I - \Lambda)^{-1}(I - \Lambda)$ is a row-stochastic matrix (i.e., a matrix with nonnegative elements, and each row summing to 1). The matrix L is the Laplacian matrix (hereafter: Laplacian) of the graph $\mathcal{G}$, defined as $L := \Delta - A$ where Δ is the in-degree matrix of $\mathcal{G}$, i.e., $\Delta = \mathrm{diag}\,(\delta_1, \ldots, \delta_n)$ with $\delta_i = \sum_{j=1}^{n} a_{ij}$ for all i. $\Lambda = \mathrm{diag}\,(\lambda_1, \ldots, \lambda_n)$ is the diagonal matrix of the susceptibility parameters. The assumption that there exists at least one agent i that stubbornly holds onto its prejudice u_i (i.e., $\lambda_i < 1$), means that the FJ model (1) will converge to disagreement, i.e., there exists at least a pair of agents i and j that will reach different opinions in time, $x_k(\infty) \neq x_j(\infty)$.

[1] For other continuous-time versions of the FJ model, such as the Taylor's model, see [6, 7].

Including the influence of a campaign to reduce the consumption of meat

To include the influence of the campaign aimed at reducing meat consumption, an additional virtual node, i.e., an influencer agent or external entity, is linked to all agents in the food network. Letting the opinion of the influencer agent set to d, we assume that the agents' prejudices in eq. (1) are affected by the external entity as follows:

$$u_i = (1 - \gamma_i)x_i(0) + \gamma_i d. \tag{2}$$

The parameter $\gamma_i \in [0, \bar{\gamma}]$ models the influence efforts of the external entity towards each agent i; in particular, when $\gamma_i = 0$ the external entity has no influence on agent i, and the opinion forming model with (2) reduces to the FJ model (1). When $\lambda_i = 0$, γ_i captures the trade-off between stubborness towards initial opinion and influence of the external entity. The maximum value $\bar{\gamma}$ represents, for instance, the maximum extent of influence that the agent i is willing to accept or can tolerate. In this work, we assume $d = 0$ to capture an external entity aiming to shift consumption towards a vegetarian diet.

Repeated campaigns to achieve the target on meat consumption

In general, achieving the desired reduction in meat consumption may prove challenging with just one campaign. To address this issue and effectively influence behavior we consider the case of repeated campaigns. Let $s = 1, 2, \ldots$ indicate the campaign instants, where the (start and end of the) s-th campaign is captured by the interval $[s-1,\ s]$, and let the influence effort of the external entity towards each agent i at campaign s be represented by a parameter $\gamma_i(s) \geq 0$. To model repeated campaigns we can extend the model (1)–(2) as follows:

$$\dot{x}_i(s,t) = \lambda_i \textstyle\sum_{j=1}^{n} a_{ij}(x_j(s,t) - x_i(s,t)) + (1-\lambda_i)(u_i(s) - x_i(s,t)) \tag{3a}$$

$$u_i(s) = (1 - \gamma_i(s))x_i(s,0) + \gamma_i(s)d, \tag{3b}$$

$$x_i(s,0) = x_i(s-1,\infty) = \lim_{t\to\infty} x_i(s-1,t) \tag{3c}$$

where $x_i(s,t)$ describes the opinion of agent i at time t and campaign s. Eq. (3a) means that during each campaign the opinion of each agent evolves according to the continuous-time FJ model, while eq. (3b) describes how the prejudices update at each campaign. Eq. (3c) indicates the concatenation rule between different campaigns, specifically that the initial opinion at a new campaign is equal to the final opinion at the previous campaign. Using the repeated campaigns model (3) under the main external influence scenario considered in the numerical simulations (i.e., broadcasting, see below), it is possible to prove that $\lim_{s\to\infty} x_i(s,\infty) = 0$. This means that after a potentially large number of campaigns, the final opinion of each agent in the food network regarding an appropriate proportion of meat consumption will reach the desired state $d = 0$.

2.2 Parameters of the opinion forming model

2.2.1 Creation of the UK population "food" network

We define the food network as an equiprobable (undirected) graph $\mathcal{G} = (\mathcal{V}, \mathcal{E}, A)$, where $\mathrm{card}(\mathcal{V}) = n$ is the total number of individuals in a population and $A = [a_{ij}] \in \mathbb{R}^{n\times n}$ is the adjacency matrix. The graph $\mathcal{G}$ has edge probability p for the entire network, i.e., $P[(i,j) \in \mathcal{E}] = p$. In general, insights into large-scale social structures could be obtained given comprehensive information regarding the population (including demographic, socio-economic, and geographic information), see e.g. [9]. In our case, for the adjacency matrix A, we adopt a simple modeling choice consistent with our limited information setting. Based on the available data, which includes 5 age groups and 3 socioeconomic status groups, we construct the food network with a block structure to reflect how membership in different age and socioeconomic status groups may lead to different levels of trust between individuals, reflecting homophily-based connections. [10] In particular, individuals belonging to the same group are linked through a positive edge of weight equal

to +1, while individuals from different groups are linked through a nonnegative edge of weight strictly less than 1. In this case, the weights are chosen according to a linearly spaced grid. The procedure is detailed as follows. After classifying individuals into 15 groups based on age and socioeconomic status, we define a group order to represent how close ideologically a pair of individuals from different groups are. In particular, this ordering is structured such that groups are ordered first by age and then by socioeconomic status. We assume a linearly spaced grid in $[0, 1]$ that is symmetric around 0.5, i.e., the 1st, 2nd, 3rd, ..., and 15th group in the order are in position 0, $1/(15-1)$, $2/(15-1)$, ..., and 1. For example, a pair of individuals who are 20 years old but have different socioeconomic status is closer ideologically to a pair of individuals being 20 and 65+ years old, respectively. We define the adjacency matrix of $\mathcal{G}$ as:

$$a_{ij} = \begin{cases} 1, & (i,j) \in \mathcal{E} \text{ and } i,j \text{ belong to the same group} \\ 1 - |\text{position of } h - \text{position of } l|, & (i,j) \in \mathcal{E} \text{ and } i,j \text{ belong to different groups } h,l \in 1,\dots,15 \end{cases}$$

2.2.2 Vegetarians, carnivores, and omnivores

In this study, we categorize individuals into three groups: Vegetarians, carnivores, and omnivores. Vegetarians are agents that in the survey (i.e., initial conditions) report zero consumption of meat (i.e., i s.t. $x_i(0) = x\text{kcal}_i(0) = 0$), carnivores are agents that report a 100% of meat consumption (i.e., i s.t. $x_i(0) = 1$), and omnivores are agents that report a diverse diet (i.e., i s.t. $x_i(0) \in (0, 1)$). Based on the survey data, there are 129 vegetarians, 31 carnivores, and 1 681 omnivores.

Throughout the manuscript, we assume that vegetarians will not be moved in their convictions by either social pressure or external influence, that is, $\lambda_i = 0$ and $\gamma_i = 0$ for every agent i that is a vegetarian. Carnivores instead are stubborn agents that cannot be influenced by social pressure only, but whose opinion can be changed through external influence; that is, $\lambda_i = 0$ and $\gamma_i \geq 0$ for every agent i that is a carnivore. Finally, omnivores are susceptible agents, whose opinion adapts to social pressure and external influence, that is, $\lambda_i > 0$ and $\gamma_i \geq 0$ for every agent i that is an omnivore. As a design choice, we select λ_i for each omnivore agent i to connect less susceptible agents with those who exhibit a strong baseline preference for meat consumption. Notably, for 75% of the agents (i.e., the third quartile), the preferred proportion of meat in their diet exceeds (approximately) 0.44. Therefore, we assign a low susceptibility ($\lambda_i \leq 0.05$) to agents whose meat consumption preference is greater than 0.44. In particular, after dividing the interval $[0.44, 1)$ into three equal subranges, we assign each agent to a group q based on their baseline meat consumption preference, ordered increasingly. For each agent i in group q ($q = 1, 2, 3$), we then draw λ_i from a uniform distribution $\mathcal{U}_{[0.04-0.01\,(q-1), 0.05-0.01\,(q-1)]}$ (note the mean decreases as q increases). A similar procedure was adopted to determine the susceptibility parameters for the other agents. In particular, after dividing the interval $(0, 0.44]$ into nine equal subranges, we assign each agent to a group $q = 1, \dots, 9$ based on their baseline meat consumption preference, ordered increasingly. For each agent i in group q, we draw λ_i from a uniform distribution, assigning the highest value (chosen to be 0.8) to the parameters associated to the middle group ($q = 5$), and decreasing the values as q moves away from the center: $\lambda_{i \in \text{ group 1 or 9}} \sim \mathcal{U}_{[0.05,0.2]}$, $\lambda_{i \in \text{group 2 or 8}} \sim \mathcal{U}_{[0.2,0.4]}$, $\lambda_{i \in \text{group 3 or 7}} \sim \mathcal{U}_{[0.4,0.6]}$, $\lambda_{i \in \text{group 4 or 6}} \sim \mathcal{U}_{[0.6,0.8]}$, $\lambda_{i \in \text{group 5}} = 0.8$. In summary, the design choice for the susceptibility parameters reflects the idea that agents with high or low baseline preferred meat consumption are less susceptible than those with a more varied diet.

2.3 External influence scenarios in the numerical simulations

In this work, we consider different settings for the external influence parameters γ_i, $i = 1, \dots, n$, where n is the total number of individuals in the UK population under investigation. To compare the different settings, we compute the time-to-target (eq. (5)) associated with the −35% and −50% targets for decreases in meat consumption.

2.3.1 Broadcasting - high vs. low influence

The first main simulation setting is that of broadcasting, or equal external influence, in time and among the agents, i.e., $\gamma_i = \bar{\gamma}$ for all $i = 1, \ldots, n$. We select two values of $\bar{\gamma}$, i.e., $\bar{\gamma} = 0.025$ and $\bar{\gamma} = 0.025/2 = 0.0125$, capturing high and low influence, respectively. These seemingly low values capture an underlying assumption that the external entity's influence is, in general, limited.

2.3.2 Sensitivity analysis

The second setting we consider in the numerical simulations is a sensitivity analysis, where we apply diverse values of influence between the individuals, i.e., $\gamma_i \neq \gamma_j$ for all $i, j = 1, \ldots, n$ (while keeping γ constant across campaigns). We consider two cases. The first is used to test heterogeneous external influence parameters, generated as uniformly or normally distributed random numbers, while the second introduces a dependency between the baseline percentage of meat consumption and the influence parameters.

Heterogeneous external influence parameters. We first use a uniform distribution centered at $\bar{\gamma}/2 = 0.0125$ (i.e., the value adopted in the low influence scenario in the broadcast setting), yielding $\gamma_i \sim U_{[0,0.025]}$ for all i. We then draw 500 independent samples, where each sample is a vector $\gamma = [\gamma_1, \ldots, \gamma_n]^T$ with elements γ_i drawn from this distribution. The time-to-target varies between approximately 9.5 and 10.4 years (with a median of 10.1 years) across the 500 samples.

To study a more general case, we also consider a truncated normal distribution with support in the range $[0, \bar{\gamma}]$, parameterized by a fixed standard deviation $\sigma = 0.2\,\bar{\gamma}$ and a mean μ that varies across 20 values. Specifically, we set $\mu \in \{0.05\,\bar{\gamma}, 0.1\,\bar{\gamma}, \ldots, 0.95\,\bar{\gamma}, \bar{\gamma}\}$. As μ decreases, the distribution becomes more concentrated near 0, while as μ increases, it becomes more spread out towards $\bar{\gamma}$. For each value of μ, we draw a vector $\gamma = [\gamma_1, \ldots, \gamma_n]^T$ with elements γ_i drawn from this truncated normal distribution, i.e., $\gamma_i \sim \mathcal{N}_{[0,\bar{\gamma}]}(\mu, \sigma)$ for all $i = 1, \ldots, n$ (Supplementary Figure 2A, left panel). Intuitively, this means that when μ is small (resp. high) the external entity has a small (resp. high) overall influence. In this case, we observe a time-to-target ranging (in median, across the 500 samples) between approximately 21.6 years (small μ) and 6.1 years (high μ) (Supplementary Figure 2A, right panel).

Dependency between baseline percentage of meat consumption and influence parameters To establish a dependency between baseline percentage of meat consumption $x(0)$ and the influence parameters, we consider two adequate orderings of samples of the influence parameters γ. We assume that these samples are drawn from the truncated normal distributions described in the previous paragraph and illustrated in Supplementary Figure 2A, that is, $\gamma = [\gamma_1, \ldots, \gamma_n]^T$ where $\gamma_i \sim \mathcal{N}_{[0,\bar{\gamma}]}(\mu, \sigma)$ for all $i = 1, \ldots, n$.

We first need to introduce the following two orderings: $o : \{1, \ldots, n\} \mapsto \{1, \ldots, n\}$ such that $x_{o(1)}(0) \leq x_{o(2)}(0) \leq \cdots \leq x_{o(n)}(0)$ and $\pi : \{1, \ldots, n\} \mapsto \{1, \ldots, n\}$ such that $\gamma_{\pi(1)} \leq \cdots \leq \gamma_{\pi(n)}$, sorting $x(0)$ and γ, respectively. To sort the values of γ in increasing order according to the baseline percentage of meat consumption, we assign $\gamma_{o(i)} = \gamma_{\pi(i)}$ for $i = 1, \ldots, n$, or, in another (perhaps easier) notation, $\gamma_{o(i)} = \text{sorted}\big(\mathcal{N}_{[0,\bar{\gamma}]}(\sigma, \mu)\big)_i$ (Supplementary Figure 2B, top-left panel). Alternatively, to sort the values of γ in decreasing order relative to the baseline percentage of meat consumption, we assign $\gamma_{o(i)} = \gamma_{n-\pi(i)+1}$ for $i = 1, \ldots, n$, i.e., $\gamma_{o(i)} = \text{sorted-decreasing}\big(\mathcal{N}_{[0,\bar{\gamma}]}(\sigma, \mu)\big)_i$ (Supplementary Figure 2B, bottom-left panel). Intuitively, the first case implies that the external entity predominantly influences individuals with a higher proportion of meat in their diet, while the second implies that the external entity has a smaller influence on those with higher meat consumption.

Comparing the two cases, we expect a longer time-to-target in the second (Supplementary Figure 2B, right panels). As expected, the number of years needed to achieve the 35% target on meat consumption changes significantly between the two cases, from approximately 17.3 to 29.1 years (small μ) and from 5.8 to 6.3 years (high μ), see right panels in Supplementary Figure 2B.

2.3.3 Budget constraints and targeted campaigns

The last settings we consider involve budget constraints and strategic decision from the external entity, who needs to select an optimal sequence strategy w.r.t. a cost function while respecting a budget constraint. As detailed in the main manuscript, we explore two situations: 1) The external entity is able to influence all agents in each campaign, but a budget constraint limits the total number of campaigns where this influence can occur; and 2) For each campaign, a budget constraint limits the total number of agents that can be influenced. Based on these situations, two questions arise: How can the external entity allocate its influence efforts in the optimal way? And how does this allocation affect the scaled average relative change? As shown in [5], the questions can be framed as problems of optimal time and space budget allocation, respectively, for a strategic external entity aiming to steer agents' opinions toward a desired state. To determine the influence weights between the external entity and the agents within the social network, i.e., the external efforts parameters γ_i in our model, we follow the approach taken in [5]; however, differently from [5] we consider prejudiced agents. Detailed derivations of the theoretical results are presented in [11] and summarized briefly in the next two sections.

Overall, we can observe that, by imposing budget constraints on broadcast influence, the average relative change (eq. (4)) ranges between -6.0% obtained in the worst-case scenario, where external influence is limited to 10% of the total number of campaigns, to -36% obtained for the high influence broadcast scenario (Supplementary Figure 3A). Similarly, by limiting the total number of individuals that can be influenced in each campaign, we note that the average relative change varies between -5.8% (only 10% of agents can be influenced) to -36% (high influence broadcast scenario). The analysis also highlights that achieving the desired target of -35% of meat consumption within 5.2 years requires the external entity to influence only 80% of individuals (Supplementary Figure 3B).

Broadcast influence with budget constraints The first strategy we consider is broadcasting across the community, i.e., $\gamma_i(k) = \gamma_j(k)$ for all $i, j = 1, \dots, n$ and $k \in \mathbb{N}$. With some abuse of notation, we let $\gamma_i(k) = \gamma(k) \in [0, \bar{\gamma}]$ for all i. In this case, we assume a budget constraint $\sum_{k=1}^{s} \gamma(k) \leq B/n$ for s campaigns. The external influence aims at selecting an optimal sequence strategy $\{\gamma(k)\}_{k=1}^{s}$ that minimizes a certain cost while respecting the budget constraint B. The cost function is selected as $J_s(\{\gamma(k)\}_{k=1}^{s}) = \frac{1}{n}\left|\mathbb{1}^T(x(s,\infty) - d)\right|$, i.e., as the average distance of the collective opinion of the network from the desired opinion d. If the budget B is large (i.e., $B \geq sn\bar{\gamma}$), then the optimal solution corresponds to selecting $\gamma(k) = \bar{\gamma}$ for all $k = 1, \dots, s$. Instead, if the budget is small (i.e., $B < sn\bar{\gamma}$), solving the constrained optimization problem corresponds to adopting the broadcasting strategy $\{\gamma(k)\}_{k=1}^{s}$ defined as:

$$\gamma(k) = \begin{cases} \bar{\gamma}, & \text{if } k \leq \lfloor \frac{B}{n\bar{\gamma}} \rfloor, \\ \frac{B}{n} - \bar{\gamma}\lfloor \frac{B}{n\bar{\gamma}} \rfloor, & \text{if } k = \lfloor \frac{B}{n\bar{\gamma}} \rfloor + 1, \\ 0, & \text{otherwise.} \end{cases}$$

which allocates the influence efforts at the earliest opportunity. The results are illustrated in Supplementary Figure 3A.

Targeted influence one step ahead with budget constraints The second strategy we consider is that of targeted campaigns, where each vector $\gamma(s)$ is designed to minimize the cost at the end of the campaign s, given the observed opinions at the end of campaign $s-1$, and under a budget constraint imposed for that specific campaign denoted by B_s. The cost function is the same as the previous case. If the budget B_s is large (i.e., $B_s \geq n$), then the optimal solution corresponds to selecting $\gamma_i(s) = \bar{\gamma}$ for all agents i. Instead, let the budget B_s satisfy $B_s < n$. Moreover, let $\rho = V^T\mathbb{1}/n$ be the social power of the individuals, where the matrix V is defined in Supplementary Section 2.1, and $c = [c_1, \dots, c_n]^T$ with $c_i = \rho_i\big(x_i(s-1) - d\big)$. Define the ordering $o : \{1, \dots, n\} \mapsto \{1, \dots, n\}$ such that $c_{o(1)} \geq c_{o(2)} \geq \dots \geq c_{o(1)}$.

In this case, solving the constrained optimization problem corresponds to adopting the targeted strategy $\gamma(s)$ defined as:

$$\gamma_{o(i)}(s) = \begin{cases} \bar{\gamma}, & \text{if } i \le \lfloor \frac{B_s}{\bar{\gamma}} \rfloor, \\ B_s - \bar{\gamma}\lfloor \frac{B_s}{\bar{\gamma}} \rfloor, & \text{if } i = \lfloor \frac{B_s}{\bar{\gamma}} \rfloor + 1, \\ 0, & \text{otherwise.} \end{cases}$$

which allocates the influence efforts prioritizing the agents that are farther from the desired target (weighted by their social power). The results are illustrated in Supplementary Figure 3B.

3 Metrics

We define three metrics to evaluate the numerical simulations.

The first indicator is the relative change at each time t w.r.t. the baseline consumption; for each individual $i = 1, \ldots, n$, it is defined as the difference between their consumption at each time t and the average consumption at initial conditions, i.e., $x\text{kcal}_i(t) - \frac{1}{n}\sum_{i=1}^{n} x\text{kcal}_i(0)$. Similarly, the average relative change is defined as the difference between the average consumption at each time t and the average consumption at initial conditions, i.e., $\frac{1}{n}\sum_{i=1}^{n} x\text{kcal}_i(t) - \frac{1}{n}\sum_{i=1}^{n} x\text{kcal}_i(0)$. To ease of interpretation we use the following scaled versions:

$$\text{rc}_i(t) = \frac{x\text{kcal}_i(t) - \frac{1}{n}\sum_{i=1}^{n} x\text{kcal}_i(0)}{\frac{1}{n}\sum_{i=1}^{n} x\text{kcal}_i(0)} \%, \tag{4a}$$

$$\text{rc}_{\text{avg}}(t) = \frac{1}{n}\sum_{i=1}^{n} \text{rc}_i(t) = \frac{\frac{1}{n}\sum_{i=1}^{n} x\text{kcal}_i(t) - \frac{1}{n}\sum_{i=1}^{n} x\text{kcal}_i(0)}{\frac{1}{n}\sum_{i=1}^{n} x\text{kcal}_i(0)} \%. \tag{4b}$$

The second indicator is the minimum time t for which the (scaled) average relative change in eq. (4) reaches the desired decrease in meat consumption ($d_{\%}$, see Supplementary Section 1). In other words, $t_{d_{\%}}$ indicates the minimum number of weeks to achieve the target on meat consumption ($d$kcal, see Supplementary Section 1).

$$t_{d_{\%}} = \min\{t \ge 0 \ \text{ s.t. } \text{rc}_{\text{avg}}(t) \le -d_{\%}\}, \quad d_{\%} \in \{35\%, 50\%\}. \tag{5}$$

The third indicator is the percentage of adopters at each time t, i.e., the percentage of agents whose meat consumption is below the desired target $d$kcal:

$$\text{adopters}(t) = \frac{\text{card}(\{i : x\text{kcal}_i(t) < d\text{kcal}\})}{n} \% \in [0\%, 100\%]. \tag{6}$$

References

[1] Climate Change Committee, "The Sixth Carbon Budget: The UK's path to Net Zero," Climate Change Committee, Tech. Rep., 2020. https://www.theccc.org.uk/publication/sixth-carbon-budget/

[2] B. Bates *et al.*, "National Diet and Nutrition Survey: Time trend and income analyses for Years 1 to 9," Tech. Rep., 2019. https://assets.publishing.service.gov.uk/media/5c45e22340f0b61704aec504/NDNS_UK_Y1-9_report.pdf

[3] Y. Tian and L. Wang, "Opinion dynamics in social networks with stubborn agents: An issue-based perspective," *Automatica*, vol. 96, pp. 213–223, 2018. https://doi.org/10.1016/j.automatica.2018.06.041

[4] L. Wang *et al.*, "Consensus in concatenated opinion dynamics with stubborn agents," *IEEE Transactions on Automatic Control*, vol. 68, no. 7, pp. 4008–4023, 7 2023. https://ieeexplore.ieee.org/document/9864235/

[5] I. Morărescu *et al.*, "Space–time budget allocation policy design for viral marketing," *Nonlinear Analysis: Hybrid Systems*, vol. 37, p. 100899, 8 2020. https://doi.org/10.1016/j.nahs.2020.100899https://linkinghub.elsevier.com/retrieve/pii/S1751570X20300467

[6] M. Taylor, "Towards a mathematical theory of influence and attitude change," *Human Relations*, vol. 21, no. 2, pp. 121–139, 5 1968. http://journals.sagepub.com/doi/10.1177/001872676802100202

[7] A. V. Proskurnikov and R. Tempo, "A tutorial on modeling and analysis of dynamic social networks. Part I," *Annual Reviews in Control*, vol. 43, pp. 65–79, 2017.

[8] C. Altafini, "Notes for a course: Opinion Dynamics in Social Networks," 2022.

[9] E. Bokányi, E. Heemskerk, and F. Takes, "The anatomy of a population-scale social network," *Scientific Reports*, vol. 13, p. 9209, 2023.

[10] N. Larson and M. Story, "A review of environmental influences on food choices," *Annals of Behavioral Medicine*, vol. 38, no. SUPPL., pp. 56–73, 2009.

[11] A. Fontan *et al.*, "Influencing opinion dynamics to promote sustainable food choices," in *5th IFAC Workshop on Cyber-Physical Human Systems*, Antalya, Turkey, 2024, pp. 1–6.

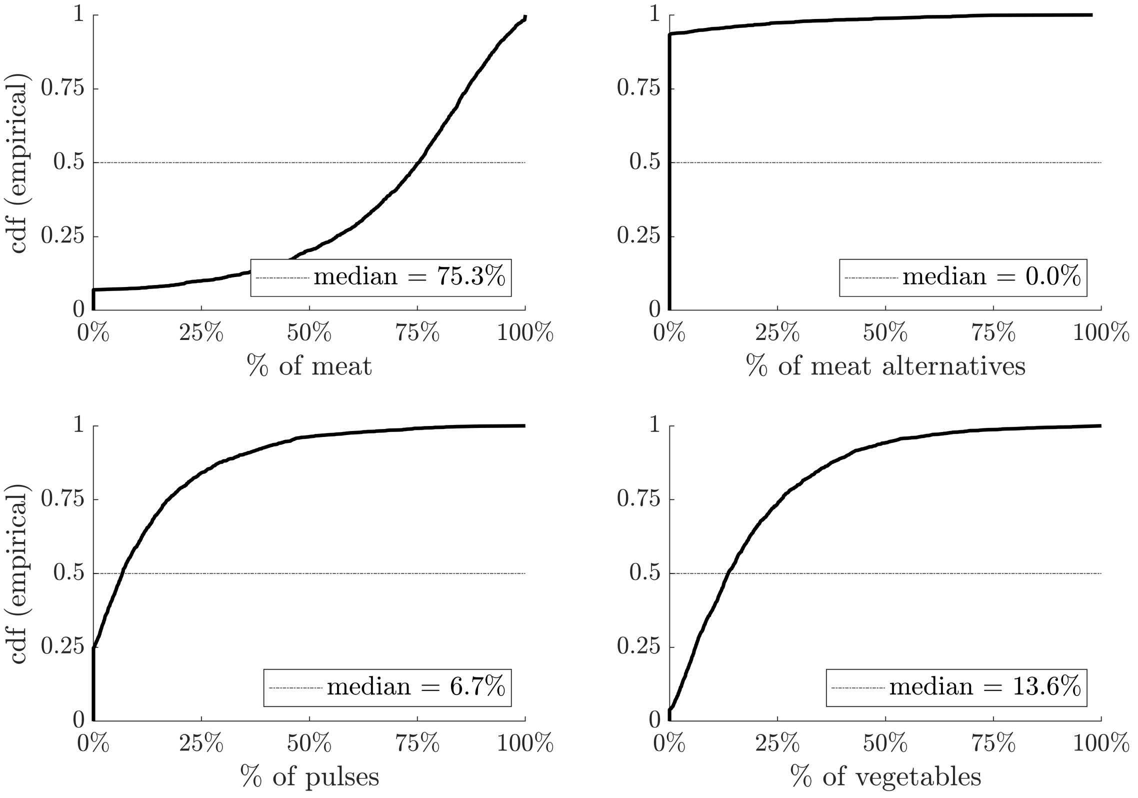

Supplementary Figure 1: Distribution of baseline consumption (i.e., consumption of food groups w.r.t. the energy intake from meat and substitutes) in the UK population, calculated using the data obtained from the National Diet and Nutrition Survey 2019 [2]. Figure 1 in the main manuscript displays the distribution in absolute values (kcal and grams). The normalization proposed here, based on the empirical cumulative distribution function (cdf), facilitates a more equitable representation and comparison of the population. Dashed-dotted lines indicate the median.

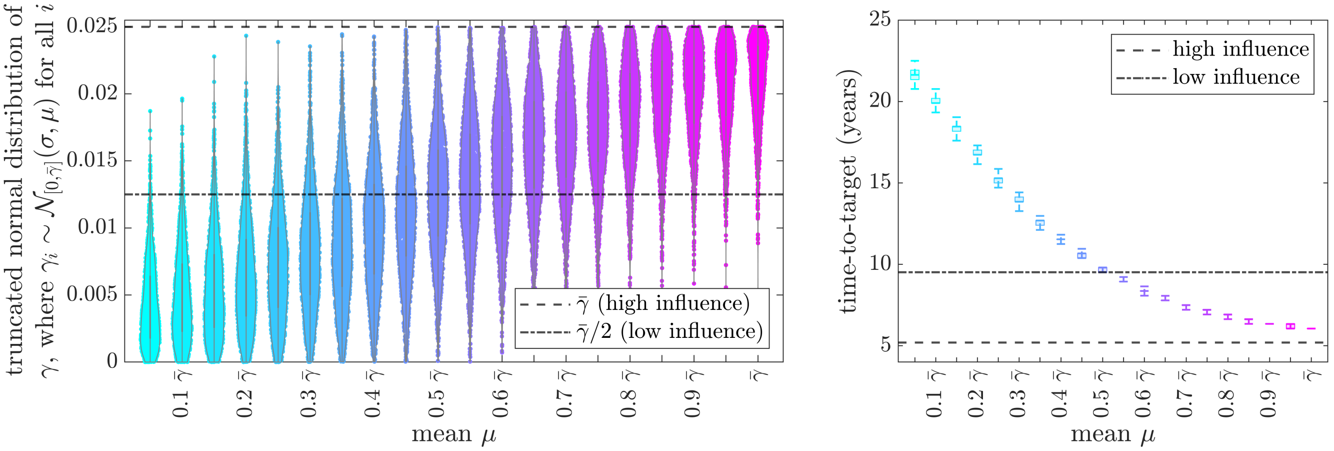

(A)

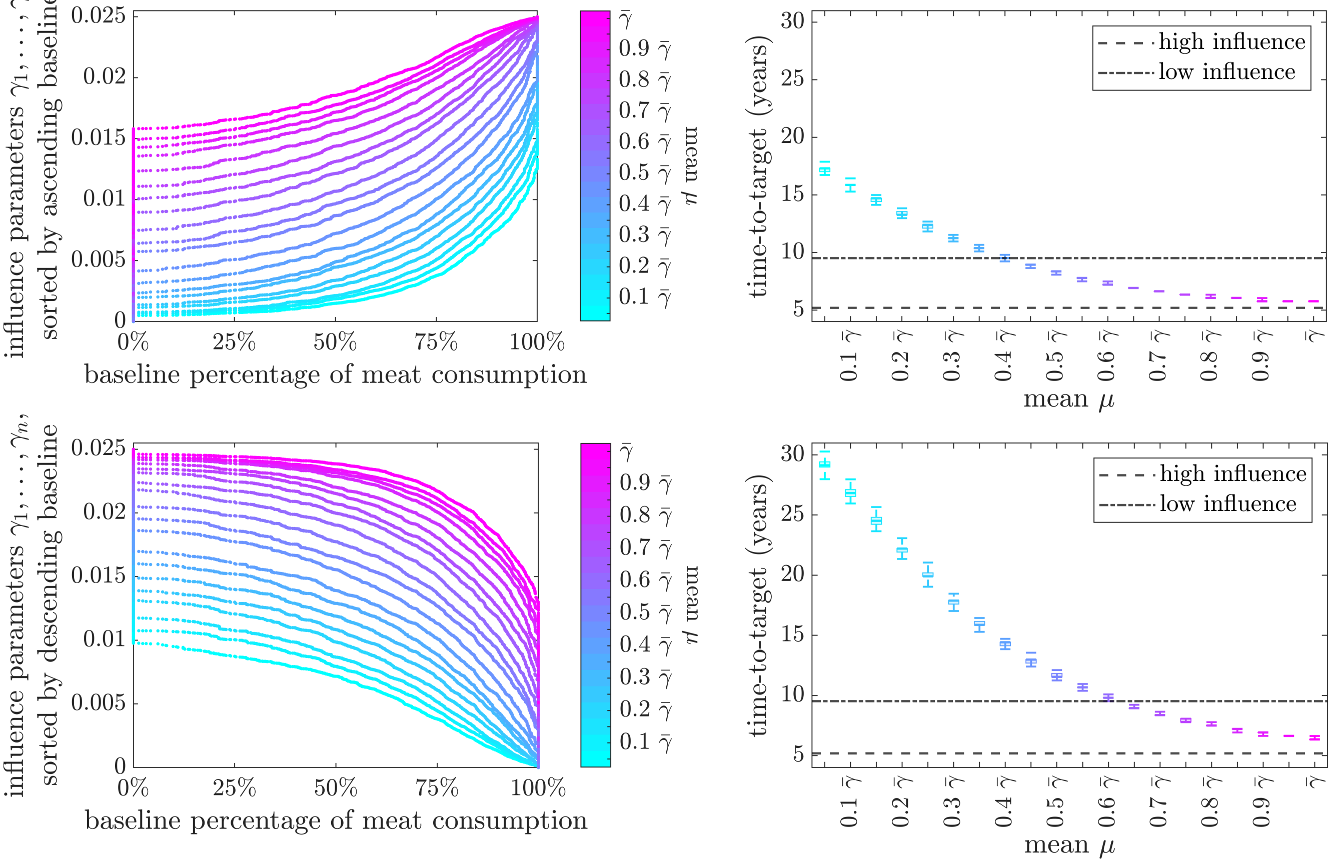

(B)

Supplementary Figure 2: Time-to-target corresponding to the −35% target for different external influence parameters. (**A**): Heterogeneous external influence parameters. The left panel shows the truncated normal distributions of $\gamma = [\gamma_1, \ldots, \gamma_n]^T$, where each γ_i is drawn from $\mathcal{N}_{[0,\bar{\gamma}]}(\mu, 0.2\bar{\gamma})$, for different values of the mean $\mu \in \{0.05\,\bar{\gamma}, 0.1\,\bar{\gamma}, \ldots, \bar{\gamma}\}$. For each μ, 500 samples of the influence parameters γ are drawn from the distribution. The right panel shows the time-to-target (eq. (5)) for each γ (for each μ, the box plots show the variability across the 500 samples). (**B**): A dependency between baseline percentage of meat consumption and influence parameters is established by sorting each sample of γ. The left panels display two orderings (for illustrative purposes, only one out of 500 samples of γ is shown for each μ): the first (top left) aligns γ with baseline meat consumption, meaning that external entity predominantly affects individuals with higher meat consumption, while the second orders γ in descending order (bottom left). The right panels show the time-to-targets for each sample of the influence parameters.

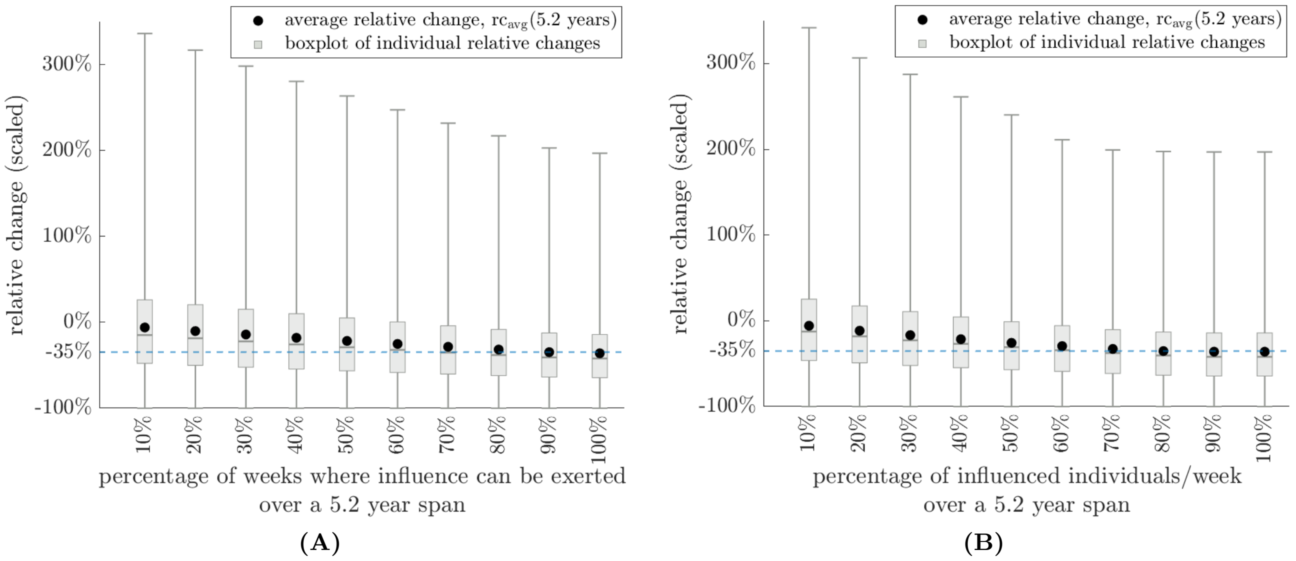

Supplementary Figure 3: Relative change (scaled) under budget constraints corresponding to the −35% target, when the external entity adopts one of the two strategies described in Supplementary Section 2.3.3. The boxplots illustrate the variability of the individual relative changes (eq. (4a)) across the agents, while the black circles indicate the average relative change (eq. (4b)). Both indicators are reported only at the 5.2 years mark, which corresponds to the time-to-target obtained in the (unconstrained) high influence broadcast scenario (see Fig 2 in the main text). A dashed line indicates the −35% target. (**A**): The external entity is able to influence all agents in each campaign, but a budget constraint limits the total number of campaigns where this influence can occur (Section 2.3.3, broadcast influence with budget constraints). (**B**): For each campaign, a budget constraint limits the total number of agents that can be influenced (Section 2.3.3, targeted influence one step ahead with budget constraints).

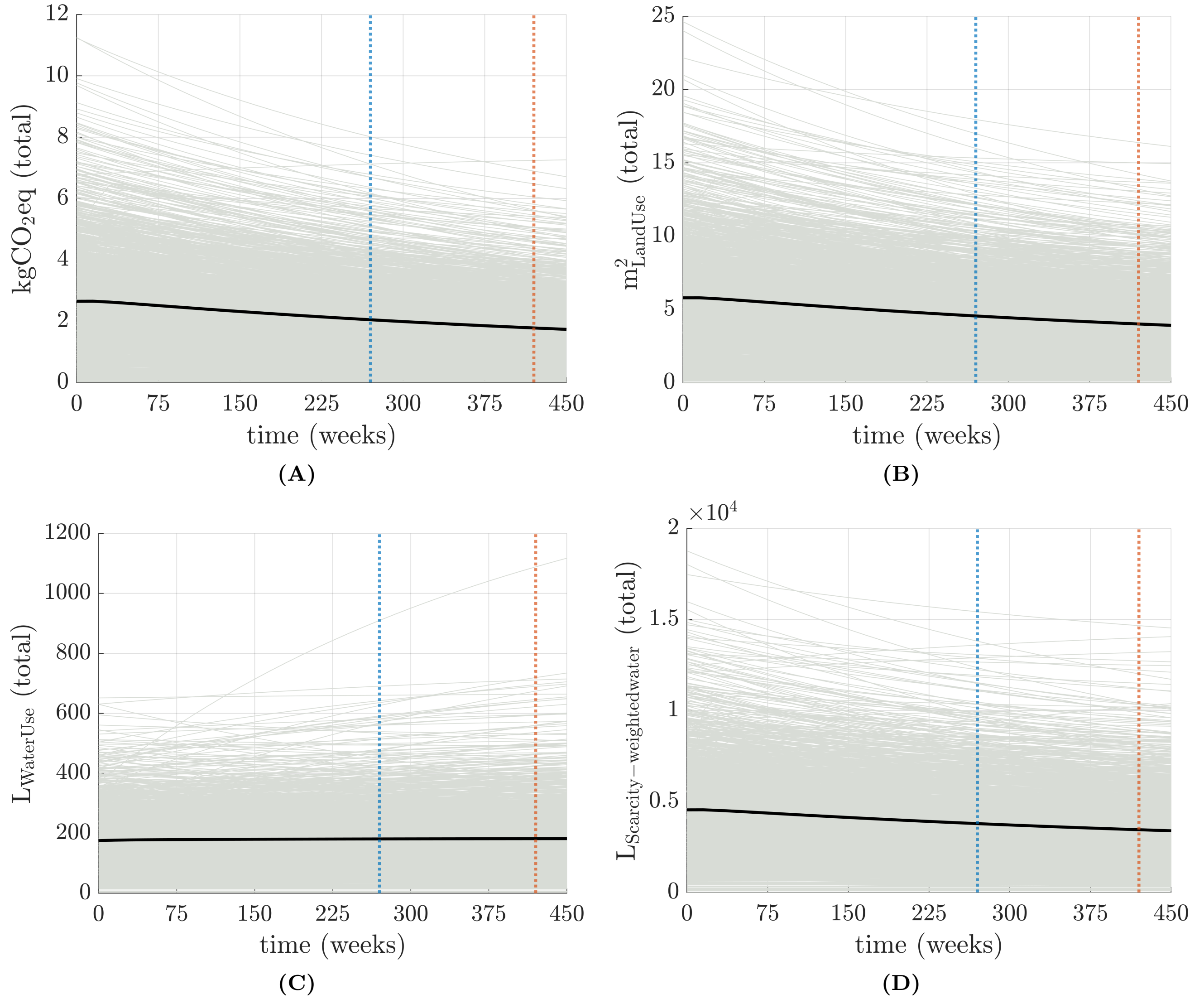

Supplementary Figure 4: Environmental impacts from Supplementary Table 2 (total impacts from meat, meat alternatives, pulses, and vegetables) during repeated campaigns to reduce the consumption of meat (high influence scenario). Gray trajectories represent individual environmental impacts (i.e., the environmental impacts associated with each individual in the population and their consumption values); black trajectories represent the average environmental impacts. Vertical dotted lines indicate the time-to-target (eq. (5)), corresponding to a 35% (blue color) and 50% (red color) decrease. (**A**): $kgCO_2eq$. (**B**): m^2_{LandUse}. (**C**): L_{WaterUse}. (**D**): $L_{\mathrm{Scarcity-weightedwater}}$.

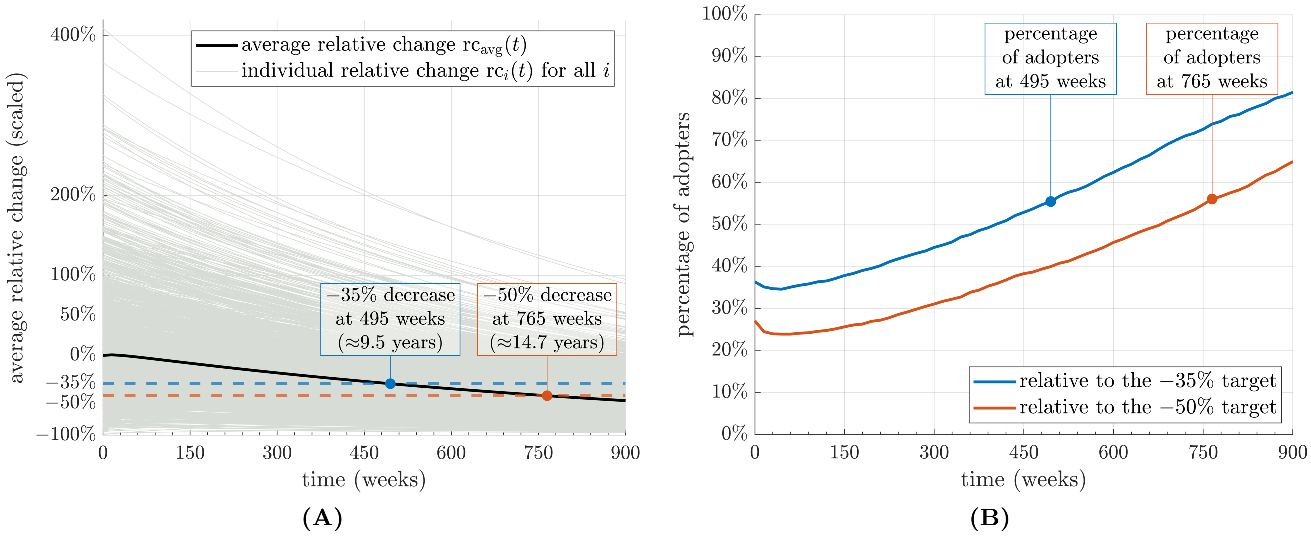

Supplementary Figure 5: Performance indicators during repeated campaigns to reduce the consumption of meat, with decrease targets equal to −35% and −50% (low influence scenario, to compare with Figure 2 in the main manuscript for the high influence scenario). (**A**): Scaled average relative change ($rc_{avg}(t)$ in eq. (4)), indicated by a thick black line, and individual relative changes across the population ($rc_i(t)$ in eq. (4)), represented by light gray lines. Dashed lines indicate the desired decrease in meat consumption and the two circles the corresponding time to target (eq. (5)). (**B**): Percentage of adopters (eq. (6)) relative to the −35% (blue line) and −50% (red line) targets.

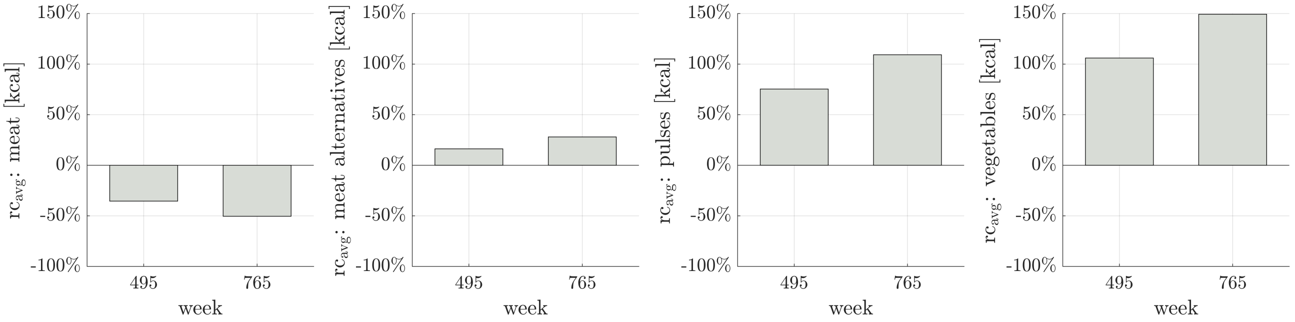

(A)

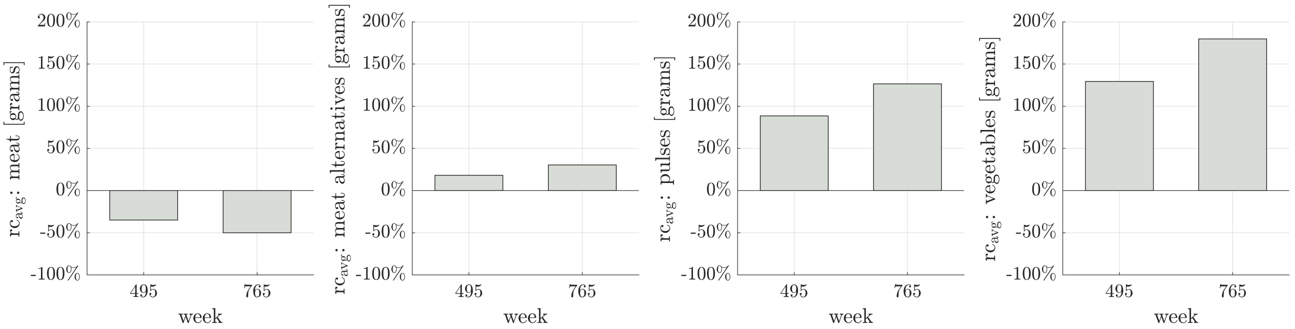

(B)

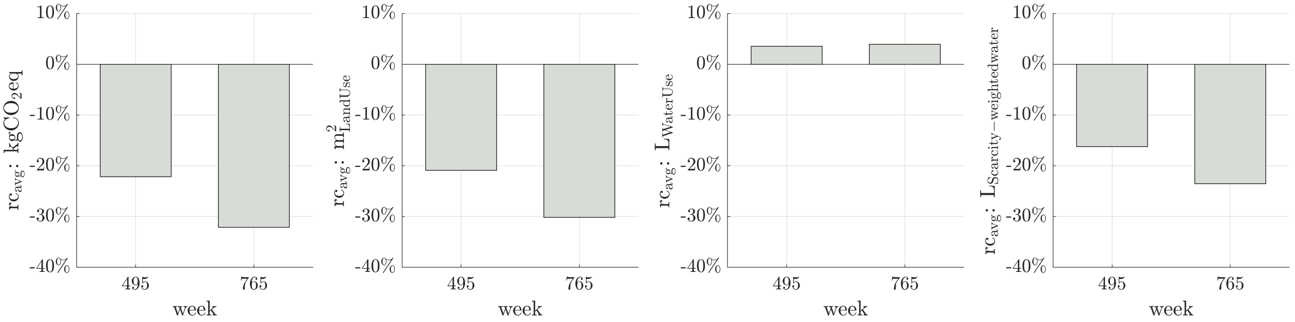

(C)

Supplementary Figure 6: Average relative change at time-to-targets (eq. (4)) corresponding to 35% and 50% targets decrease of meat consumption, respectively (low influence scenario, to compare with Figure 3 in the main manuscript for the high influence scenario). (**A**): For the consumption of all food groups in kcal. (**B**): For the consumption of all food groups in grams. (**C**): For the environmental impacts from Supplementary Table 2.

Simulated food group	NDNS Food group
Meat	Bacon and ham, Burgers and kebabs, Chicken and turkey dishes, Coated chicken, Lamb and dishes, Liver and dishes, Other meat and meat products, Pork and dishes, Sausages, Beef veal and dishes
Meat alternatives	Meat alternatives including ready meals and homemade dishes
Pulses	Beans and pulses including ready meal and homemade dishes, Baked beans, Green beans not raw, Peas not raw
Vegetables	Carrots raw, Carrots not raw, Leafy green vegetables not raw, Tomatoes raw, Tomatoes not raw, Other vegetables including homemade dishes, Salad and other raw vegetables

Supplementary Table 1: Four food groups (meat, meat alternatives, pulses, vegetables) used in the numerical simulations and corresponding NDNS subfood groups [2].

	Meat	Meat Alternatives	Pulses	Vegetables
$\mathrm{kgCO_2eq/100g\ food}$	2.26	0.12	0.13	0.12
$\mathrm{m^2_{LandUse}/100g\ food}$	4.85	0.34	0.92	0.17
$\mathrm{L_{WaterUse}/100g\ food}$	123	23	21	25
$\mathrm{L_{Scarcity-weightedwater}/100g\ food}$	3636	1143	1055	1547

Supplementary Table 2: Values for the environmental impacts per 100g of food: GHGE (CO_2), land use, water use, and scarcity-weighted water use.

Consumption [grams]	Males mean	Males SD	Females mean	Females SD
**Meat**				
Baseline consumption	129.9	84.5	91.6	64.9
At week 270 (≈ 5.2 years)	83.9	49.5	62.0	39.3
At week 420 (≈ 8.1 years)	63.7	37.2	47.4	29.7
**Meat alternatives**				
Baseline consumption	2.6	12.3	2.7	13.6
At week 270 (≈ 5.2 years)	3.2	13.8	3.1	14.0
At week 420 (≈ 8.1 years)	3.6	15.1	3.4	15.2
**Pulses**				
Baseline consumption	41.7	51.5	33.0	39.3
At week 270 (≈ 5.2 years)	86.4	92.2	55.1	59.0
At week 420 (≈ 8.1 years)	106.0	115.0	66.0	71.6
**Vegetables**				
Baseline consumption	135.6	110.5	143.9	113.7
At week 270 (≈ 5.2 years)	344.3	277.9	294.0	210.5
At week 420 (≈ 8.1 years)	429.7	364.1	360.6	272.3

(a)

Environmental impacts	Males mean	Males SD	Females mean	Females SD
**$kgCO_2eq$**				
Baseline impact	3.2	1.9	2.3	1.5
At week 270 (≈ 5.2 years)	2.4	1.3	1.8	1.1
At week 420 (≈ 8.1 years)	2.1	1.2	1.6	0.9
**m^2_{LandUse}**				
Baseline impact	6.9	4.1	5.0	3.1
At week 270 (≈ 5.2 years)	5.5	3.0	4.0	2.3
At week 420 (≈ 8.1 years)	4.8	2.7	3.5	2.0
**L_{WaterUse}**				
Baseline impact	203.0	107.1	156.2	82.5
At week 270 (≈ 5.2 years)	208.1 (+2.5%)	114.4	162.1 (+3.8%)	88.4
At week 420 (≈ 8.1 years)	208.8 (+2.9%)	121.8	163.1 (+4.4%)	92.9
**$L_{\mathrm{Scarcity-weightedwater}}$**				
Baseline impact	5400.6	3071.9	3932.5	2331.0
At week 270 (≈ 5.2 years)	4528.3	2471.9	3325.4	1825.9
At week 420 (≈ 8.1 years)	4136.4	2300.1	3014.6	1647.6

(b)

Supplementary Table 3: Absolute change between baseline impact and numerical simulation at the time-to-targets $t_{35\%} = 270$ weeks and $t_{50\%} = 420$ weeks (eq. (5)) associated with desired percentage decreases equal to -35% and -50%, respectively. In contrast with Table 1 in the main manuscript, here the simulations use different fixed proportions of meat alternatives, pulses, and vegetables (i.e., parameters $\alpha_{i,\text{meat alternatives}}$, $\alpha_{i,\text{pulses}}$, $\alpha_{i,\text{vegetables}}$ for each agent i, see Supplementary Section 2). (**A**): Consumption of food groups in grams. (**B**): Environmental impacts from Table 2 (total impacts from meat, meat alternatives, pulses, and vegetables). The increased environmental impact of water use (compared to Table 1 in the main manuscript) is indicated by the corresponding percentage inside the parentheses.
